# Synthetic Multivalent Antifungal Peptides Effective against Fungi

**DOI:** 10.1371/journal.pone.0087730

**Published:** 2014-02-03

**Authors:** Rajamani Lakshminarayanan, Shouping Liu, Jianguo Li, Muruganantham Nandhakumar, Thet Tun Aung, Eunice Goh, Jamie Ya Ting Chang, Padhmanaban Saraswathi, Charles Tang, Siti Radiah Binte Safie, Lim Yih Lin, Howard Riezman, Zhou Lei, Chandra S. Verma, Roger W. Beuerman

**Affiliations:** 1 Singapore Eye Research Institute, Singapore, Singapore; 2 Department of Neuroscience and Behavioural Disorders, Duke-NUS Graduate Medical School, Singapore, Singapore; 3 Bioinformatics Institute (A*STAR), Singapore, Singapore; 4 Department of Biochemistry and NCCR Chemical Biology, University of Geneva, Geneva, Switzerland; 5 Department of Pathology, Singapore General Hospital, Singapore, Singapore; 6 School of Biological Sciences, Nanyang Technological University, Singapore, Singapore; 7 Department of Biological Sciences, National University of Singapore, Singapore, Singapore; Aligarh Muslim University, India

## Abstract

Taking advantage of the cluster effect observed in multivalent peptides, this work describes antifungal activity and possible mechanism of action of tetravalent peptide (B4010) which carries 4 copies of the sequence RGRKVVRR through a branched lysine core. B4010 displayed better antifungal properties than natamycin and amphotericin B. The peptide retained significant activity in the presence of monovalent/divalent cations, trypsin and serum and tear fluid. Moreover, B4010 is non-haemolytic and non-toxic to mice by intraperitoneal (200 mg/kg) or intravenous (100 mg/kg) routes. *S. cerevisiae* mutant strains with altered membrane sterol structures and composition showed hyper senstivity to B4010. The peptide had no affinity for cell wall polysaccharides and caused rapid dissipation of membrane potential and release of vital ions and ATP when treated with *C. albicans*. We demonstrate that additives which alter the membrane potential or membrane rigidity protect *C. albicans* from B4010-induced lethality. Calcein release assay and molecular dynamics simulations showed that the peptide preferentially binds to mixed bilayer containing ergosterol over phophotidylcholine-cholesterol bilayers. The studies further suggested that the first arginine is important for mediating peptide-bilayer interactions. Replacing the first arginine led to a 2–4 fold decrease in antifungal activities and reduced membrane disruption properties. The combined *in silico* and *in vitro* approach should facilitate rational design of new tetravalent antifungal peptides.

## Introduction

Resistance to current antifungal drugs has become common in recent years, mainly due to increased number of immuno-compromised patients, and has underscored the need for new classes of antifungals [1], [2]. Pathogenic fungal infections are the 7^th^ most common cause of infection-related deaths in the USA [3]. In fact, recent surveys suggest that fungal diseases are posing a growing threat to the global biota [4]. The number of available antifungals is limited and developing new antifungal drugs is challenging since common drug targets in fungi are homologues of similar molecular types in human which might also be inhibited. Among the five different classes of antifungals polyenes, azoles and allylamines target ergosterol or ergosterol biosynthetic pathways whereas 5-fluorocytosine (5-FC) targets DNA synthesis and caspofungins inhibit β-glucan synthase [5]. The extent of drug resistance also varies, as resistance to 5-FC and azoles are more common when compared to polyenes, although there are now many reports of resistance to polyenes as well [6], [7].

Since current antifungals have limited number of targets and increasing reports of resistance to the available drugs, peptide-based antifungals, which disable the membrane physiology, offer an important opportunity for rational drug design [8]–[10]. However, their antimicrobial properties are antagonized by physiological concentration of salts and polyanionic polymers (e.g., DNA, glycosaminoglycans and mucins) as well as being rapidly degraded by proteolytic enzymes in complex biological milieu, thus limiting their therapeutic potential [11], [12]. Several different strategies have been attempted to circumvent the drawbacks of antimicrobial peptides [13]–[17]. The development of multivalent peptides by assembling multiple copies of monomeric peptides around a core molecule has attracted significant interest as a new molecular phenotype for potential therapeutic use [18]–[20]. However, the antimicrobial properties of multivalent peptides have been limited to Gram-negative and/or Gram-positive bacteria and their utility as potent antifungals has not been explored in great detail [21]–[24].

We have previously reported the properties of a 10-residue peptide (RGRKVVRRKK) which showed potent activity against *P. aeruginosa,* but poor activity against fungi [25]. The anti-pseudomonas activity was attributed to the concentration-dependent formation of a non-covalent dimer in buffer as well as in liposome model of a prokaryotic membrane [26]. The present work extends the analysis to higher order covalently linked peptides. The antifungal properties of a potent tetravalent peptide (B4010) have been examined in physiological concentrations of salts and in complex biological fluids. We have also assessed the biocompatibility of B4010 including its effect on corneal reepithelialization in rabbit and systemic toxicity in mice. The antifungal action of this molecule on fungal membrane was ascertained using environmental-sensitive fluorescent probes. Finally, we demonstrate that coupling MD simulations with in vitro experiments shed light on the important residues that are essential for mediating membrane-lipid interactions and rationalization of the antifungal activity.

## Materials and Methods

### Ethics Statement

All animals used in this study were treated in agreement with the tenets of the Association for Research in Vision and Ophthalmology (ARVO) Statement for the Use of Animals in Ophthalmic and Vision Research, and the protocol was approved by the Singhealth Institutional Animal Care and Use Committee (IACUC; AALAC accredited, #2012/SHS/775).

### Chemicals and Peptides

Sabouraud’s Dextrose Agar was purchased from Acumedia (Michigan, MI, USA). Peptides were purchased from EZBiolabs Inc., (Carmel, IN, USA). Lipids such as L-α-phophatidylcholine (PC), L-α-phostidylethanolamine (PE), L-α-phosphatidylserine (PS) and L-α-phosphatidylinositol (PI) were bought from Avanti Polar Lipids Inc. (AL, USA). Ergosterol, sodium azide (NaN3), Carbonyl cyanide m-chloro phenylhydrazone (CCCP), 4-aminopyridine (4-AP), 5-nitro-2-(3-phenylpropylamino) benzoic acid (NPPB), Gadolinium III Chloride, tetra ethyl ammonium chloride (TEA) and 3′,3′-dipropylthiadicarbocyanine (diS-C3-5) dye were purchased from Sigma-Aldrich Corp. (MO, USA). ATP bioluminescence kit was obtained from Molecular Probes Inc (OR, USA). Amphotericin B and Natamycin were obtained in powder form from Sigma-Aldrich (S) Pte Ltd (Singapore).

### Determination of Minimum Inhibitory Concentration (MIC)

The yeast strains were cultivated and suspended in Sabouraud’s Dextrose (SD) broth diluted to one sixth at a starting OD_600_ =  ∼0.08 in a flat-bottomed microtitre plate. A serial dilution of peptide in the same broth was mixed with the inoculum to give a final peptide concentration of 0.4–22 µM. The antifungal activity was assessed by monitoring the OD_600_ in cycles of 30 minutes and an orbital shaking at 100 rpm using an Infinite M200 microplate reader (Tecan Group Ltd., Switzerland) for 24/48 h at 37°C. Cultures without peptides were used as positive controls and broth alone or with 22 µM peptide served as negative controls. The minimum concentration required for complete inhibition was assessed by both visible observations as well as by measuring the OD_600_ and taken as the MIC. Each experiment was repeated in triplicates. For studying the effects of metal ions, SD broth was adjusted with appropriate concentrations of salts to yield a final concentration of 100 and 135 mM for NaCl and KCl and 0.5–2 mM for CaCl_2_ and MgCl_2_. MIC determinations were carried out as described above. *C. albicans* ATCC10231 cells and salts in broth were taken as a positive control. Salts alone (NaCl, KCl, and CaCl_2_ and MgCl_2_) in broth or with 22 µM peptide served as a negative control. The effect of 5% human serum on the activities of B4010 against two *C. albicans* clinical isolates was determined. The human male serum was centrifuged at 13,000 rpm for 10 mins in order to remove the lipids and the supernatant was collected [27]. The MIC values against clinical isolates of *C. albicans* 2672R and *C. albicans* 1976R were determined both in standard medium (SD broth) as well as in standard medium containing 5% serum supernatant as before.

### Determination of MIC against Wild Type (WT) and Mutant *S. cerevisiae* Strains

A few identical colonies from WT and mutant strains were immediately inoculated and grown overnight in SD broth containing 0.1 mg/mL ampicillin at 30°C [28], [29]. Cells were harvested by centrifugation, washed with sterile water and frozen. Cells were plated again on SD agar plates and incubated at 37°C for 48 h. Two or three identical colonies of the mutant strains were collected and inoculated in SD broth. Peptides were dissolved in sterile water and mixed with WT and mutant strains and incubated at 37°C for 48 or 72 h in a test tube. The lowest concentration of the peptide that inhibited the growth of the yeast strain was determined by visual inspection.

### Time-kill Kinetics Assay

The time-kill kinetics of B4010 was determined against two strains of *C. albicans* (ATCC 10231 and DF2672R). The cultures were grown overnight in SD broth and the cell concentration was adjusted to 10^5^–10^6^ CFU/mL with phosphate buffer. The peptide or antifungals were added to the individual cultures. Appropriate final concentrations of B4010 (1.4–11 µM for ATCC and 0.37–3.7 µM for 2672R) or amphotericin B (0.55–11 µM for 2672R strains) and natamycin (4.7–75 µM for ATCC and 30 µM for 2672R strains) were adjusted for the inocula. The test solution was incubated at 37°C with constant shaking. 100 µL of the suspension was withdrawn at predetermined time points, serially diluted (10^2^ or 10^3^ fold) and poured into the SDA plate. The plate was incubated for 48 h at 37°C for colony counting. The data were expressed in terms of % fungal viability relative to the positive control.

### Antifungal Activity of B4010 in Presence of Trypsin and 50% Tear Fluid (TF)

For all the experiments, unless otherwise stated, *C. albicans* ATCC 10231 strain was used. B4010 (1 mg/mL) was incubated with trypsin (enzyme:peptide ratio 1∶100) at 37°C. 20 µL aliquot of this mixture was withdrawn at various time intervals (0.5, 1, 2, 4 and 6 h) and mixed with 1 µL of trypsin inhibitor. The antifungal activity of the peptide was determined by adding this mixture to 180 µL of inoculum and monitoring the growth at OD_600_ for 24 h. Similar experiments performed in the presence of trypsin/trypsin inhibitor (without B4010) were used as a positive control. For assessing the antifungal activity in TF, the peptide was dissolved in freshly collected rabbit TF and incubated at 37°C for 6 h. After incubation, an equal volume of an overnight culture of *C. albicans* (∼10^6^ CFU/mL) was mixed with peptides in tear fluid and incubated at 37°C for 24 h. The final concentrations of the peptide were at 4.4, 8.8 and 22 µM. A 100 µL aliquot of serial dilutions (10^2^ or 10^3^ times) of this mixture were inoculated on a SD agar plate and then incubated for 48 h at 37°C. Culture alone and culture with 50% tear fluid served as the positive control. The data was expressed in % killing with respect to culture without tear fluid. About 6–10% killing was observed in the presence of 50% tear fluid.

### Haemolytic Assay

Haemolytic activity of peptides and antifungal drugs were determined against rabbit red blood cells [30]. Briefly, serial dilution of peptides/antifungals in PBS was mixed with rRBC (final concentration 4% v/v), incubated at 37°C for 1 h and centrifuged at 3000 rpm for 10 minutes. The release of hemoglobin in the supernatant was monitored by measuring the hemoglobin absorbance at 576 nm. The readings from cell suspension in PBS (without any additives) or 1% Triton-X100 were used as 0% or 100% haemolysis.

### Cytotoxicity on Human Conjunctival Epithelial Cells

Immortalized normal human conjunctival epithelial cells (IOBANHC) were a gift from Yolanda Diebold at the University of Valladolid, Spain [31]. IOBA-NHC cells were cultured under standard conditions (humidified atmosphere of 5% CO_2_ at 37°C) in DMEM/F12 supplemented with 1 µg/ml bovine pancreas insulin, 2 ng/ml mouse epidermal growth factor, 0.1 µg/ml cholera toxin, 5 µg/ml hydrocortisone, 10% fetal bovine serum (FBS), 50 UI/ml penicillin, and 50 UI/ml streptomycin. Cells from passages 50–80 were used in all experiments. Every day, normal culture development was observed by phase-contrast microscopy. Cells were removed by gentle trypsin incubation at confluence and counted. They were seeded into 96 well culture plates (Corning, Schiphol-Rijk, The Netherlands) for microtitration analysis of flow cytotoxicity assay (∼10,000 cells/well). Cultures were kept at 37°C for 24 h. Subconfluent cells (culture surface covering nearly 70%) were then exposed to various peptide concentrations (0.22–225 µM). Cytotoxicity was measured periodically using MultiTox-Fluor Multiplex assay kit (Promega, WI, USA) by measuring the AFC fluorescence (λ_ex_ = 485 and λ_em_ = 520 nm). We report the cell viability after 24 h incubation of the cells with the peptides since there was no detectable toxicity even after 8 h of incubation.

### Rabbit Model of Corneal wound Healing

Four New Zealand white rabbits were divided into two groups, two each for control (saline) and two each for the study group treated with B4010. Rabbits were tranquilized by intra muscular injection of 1 mL of ketamine (100 mg/mL) and 0.5 mL of xylazil (20 mg/mL). Corneas were anesthetized by topical administration of 1% xylocaine. A 5-mm trephine was used to outline the wound margin and mechanical removal of epithelial cells was carried out by sterile mini blade (BD-Beaver) leaving the basal lamina intact as previously described [32]. The animals were treated by topical administration of 22 µM of B4010 at 3 times/day. Cornea wound was visualized by staining with fluorescein sodium, which is used in the ophthalmology clinic as a non-toxic dye to disclose wounds of the cornea, and using a cobalt-blue filter with slit lamp biomicroscopy. Measurements of the residual wound area were performed during the reepithelialization process by Image-J 1.440.

### *In vivo* Toxicity

Acute toxicity of B4010 was assessed with C57BL6 (6–8 weeks old) wild type mice. Two healthy wild type mice were chosen for each routes of administration. B4010 was delivered through intra-peritoneal (200 mg/kg) or (100 mg/kg) intra-venous routes. Two animals were used for each administration and monitored through 24/48 h to determine mortality, or signs of toxicity.

### Candidacidal Activity of B4010 in Presence of Metal Ions, Energy Poisons and Ion Channel Inhibitors

To study the effects of metal ions, the broth solution containing *C. albicans* (OD_600_ = 0.4–0.6) was adjusted to appropriate final concentrations of metal ions, incubated with 5.5 µM of B4010 for 6 h and the cell viability was determined as before after 48 h. For the effects of energy poisons and ion channel inhibitors, yeast cells (10^5^–10^6^ CFU/mL) were incubated for 2 h with or without additives at 37°C. A final concentration of 5.5 µM B4010 peptide was added to the cell suspension and incubated further for another 1.5 h at 37°C. Fungal viability was obtained by counting the number of colonies formed in each plate after 48 h incubation at 37°C. SDA plates with additives (and no peptide) served as the positive control. The final concentrations of the additives were: CCCP (5 µM), NaN_3_ (5 mM), 4-AP (1 mM), NPPB (0.5 mM), Gadolinium II chloride (1 mM) and tetra ethyl ammonium chloride (15 mM). The reported values were averages of two independent duplicate experiments. Control experiments with all the additives were also performed to assess the toxicity of the additives to *C. albicans*.

### SDS-PAGE Pull down Assay

B4010 (5.5–22 µM) was incubated with chitin (from shrimp shells) or β-D-glucan for 2 h at 37°C. The mixture was centrifuged at 10,000 g and the supernatant was analyzed by SDS-PAGE (4–20%). A control experiment without chitin/β-D-glucan was also performed to quantify peptide binding to the carbohydrate polymers.

### DiS-C_3_-5 Membrane Depolarization and SYTOX Green (SG) uptake Assays

Changes in membrane potential of *C. albicans* upon addition of B4010 were monitored by the release of potential sensitive probe diS-C_3_-5 dye. Briefly, 1 ml of overnight grown mid-log phase *Candida* cells in 5 mM HEPES buffer (pH 7.0) was mixed with 10 µM diS-C_3_-5 and incubated at 37°C for 1–2 h in a thermo shaker. 800 µL of the dye-loaded cell suspension was transferred to a stirred quartz cuvette and the change in fluorescence intensity was monitored at an emission wavelength (λ_em_) of 670 nm (excitation wavelength, 622 nm) using a Quanta Master spectrofluorimeter (Photon Technology International, NJ, USA). The excitation and emission bandwidths were set at 1 and 2 nm, respectively. Once a constant fluorescence level was achieved, 10 µL of concentrated peptide solution in HEPES buffer was added so that the final concentration of peptide was 0.22 - 22 µM. The change in fluorescence intensity was monitored continuously for 1 or 2 h. To study the effects of energy poisons and ion-channel inhibitors, the additives were added prior to the addition of 5.5 µM B4010. The change in fluorescence intensity was monitored as before.

For SG uptake assay, overnight cultures of *Candida* were harvested by centrifugation, washed three times with HEPES buffer and resuspended in the same buffer. The cells were incubated with 1 µM SG in the dark prior to the addition of peptide. The increase in emission intensity at 520 nm was monitored after addition of various concentrations of peptide (λ_ex_ = 485 nm). 1% Triton-X100 was added at the end of the experiment to determine the %uptake.

### Measurement of Extracellular Cations

Overnight cultured late logarithmic phase *C. albicans* was harvested by centrifugation, washed 5 times with 10 mM HEPES (pH 7.0), resuspended in the same buffer and adjusted to an OD_600_ = 0.4. To 5 mL of this suspension, B4010 (final concentration 5.5 µM) was added and incubated at 37°C for 2 h. The mixture was centrifuged at 3,000 g and the presence of K^+^, Ca^2+^ and Mg^2+^ in the supernatant was estimated by Perkin Elmer Dual-view Optima 5300 DV inductively coupled plasma – optical emission spectrometry (Massachusetts, USA) available at CMMAC facilities (Department of Chemistry, National University of Singapore).

### ATP Bioluminescence Assay

The extracellular ATP levels upon challenging *C. albicans* with B4010 were determined as reported before [33]. Cells (OD_600_≈0.6) were incubated with or without various additives for 2 hours at 37°C with orbital shaking. B4010 (5.5 µM) was added and incubated for another 1.5 h at 37°C with shaking. Each tube was then centrifuged at 5,000 g for 5 mins. Then 225 µL of boiling TE buffer (50 mM Tris, 2 mM EDTA, pH 7.8) was added to the 25 µl of the supernatant and mixed well. This mixture was boiled again for another 2 minutes and stored at 4°C until further examination. 100 µL of a luciferin-luciferase ATP assay mixture was added to 100 µL of the supernatant and luminescence was monitored using the Infinite M200 microplate reader (Tecan Group Ltd., Switzerland). For the time-course experiment, cells (OD_600_ = 0.4) were treated with 5.5 µM peptide for various time intervals. For dose-dependent studies, the concentration of the peptide was varied from 0.4–44 µM. The extracellular ATP concentration was determined from calibration curve obtained by ATP assay kit (Molecular Probes, OR, USA) as per the manufacturer’s instruction.

### Circular Dichroism (CD) Spectropolarimetry

Far UV-CD spectra of the peptides (0.6 mg/mL) were recorded on a JASCO J810 spectropolarimeter (JASCO, Tokyo, Japan) in 10 mM PBS (pH 7.0) using a 0.1 cm path length quartz cuvette at 25 °C. Spectra were recorded from 260 nm to 190 nm in 0.1 nm steps at a scan rate of 50 nm/min. The final spectrum is the average of 4 scans. The CD data is expressed as mean residual ellipticity ([θ]_mrw_, deg cm^2^ dmol^−1^).

### Calcein Leakage Assay

Small unilamellar vesicles (SUVs) were prepared using PC/PE/PI or PS lipids (5∶2.5∶2.5) containing 15 wt% ergosterol or PC/Cholesterol (10∶1) [17]. For the preparation of PC/PE/PI or PS/Erg SUV, the lipids were dissolved in chloroform/methanol (2∶1, v/v) in a glass tube. Ergosterol was dissolved in same solvent and added to lipid mixture to make final ergosterol content of 15 wt%. The Lipid-ergosterol mixture was dried using nitrogen gas to form lipid layer. The film was hydrated in a buffer containing 20 mM PBS (pH 7), vortexed and sonicated. Each cycle of sonication (5 sec, 40°C) was followed by freezing in liquid nitrogen and thawing on a water bath kept at 37°C. The procedure was repeated 5–6 times until an optically clear dispersion appeared. The SUV was divided into two parts. To the first part 50 mM calcein was added and incubated for 1 h. The excess calcein was removed by injecting 100 µL of the calcien-loaded SUV’s into a Ultrahydrogel™ 250 (7.8 mm × 300 mm) column equilibrated with 20 mM PBS (pH 7.0) and eluted isochratically at a flow rate of 1 mL/min for 1.5 column volumes. The calcein-loaded liposomes were mixed with calcein-free liposome (1∶1) to adjust final liposome concentration. Peptide concentration was added to obtain 1∶30 or 1∶15 peptide:liposome ratio. Changes in the fluorescence signal after addition of peptide or Triton X-100 was measured on a PTI spectrofluorometer, using an excitation wavelength of 480 nm and emission wavelength of 512 nm. The Percentage calcein released at each time point was calculated using the equation,
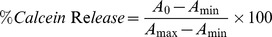



Where A_0_ is the observed fluorescence intensity upon addition of peptides, A_min_ is the average intensity at the baseline (before peptide addition), A_max_ is the average intensity at the saturation phase after adding 1% Triton X-100. Similar protocol was followed for the preparation of PC/Cholesterol SUVs.

### Isothermal Titration Calorimetry (ITC)

ITC experiments were performed using a Microcal VP-ITC Calorimeter (Microcal, Northampton, MA, USA). SUVs, B4010, and polyene antifungals were dissolved in 10 mM HEPES buffered saline at pH 7.2. SUV (PC containing 15% ergosterol) was prepared as before in the same buffer. The peptide (20 µM) or antifungal solution (natamycin at 10 µM and amphotericin B at 5 µM) was placed in the calorimetric cell (V = 1.4 mL) and the lipid vesicles (300 µM) were taken in the syringe. Aliquots of 7 µL of liposome (300 µM for B4010/natamycin and 75 µM for amphotericin B) were injected into the cell with constant stirring (310 rpm) at 25°C. A total of 40 injections were performed and the heat of reaction produced by each injection was determined by integration of heat flow traces. In a separate experiment, the heat of dilution was determined by titrating SUVs into the buffer solution (without peptide). The heat of dilution was subtracted from the heat changes determined in the peptide/antifungal-liposome experiments.

### Molecular Dynamics Simulation Studies

Molecular dynamics simulations were used to study the mode of interaction of B4010 with a fungal model membrane. Fungal membranes have complicated lipid compositions such as PS/PI, PC and PE and ergosterol. We constructed a model fungal membrane consisting of 288 lipids with four typical lipids: POPC, POPE, POPS, and erogosterol, at a ratio of 4∶2:1∶2. The CHARMM force field was used to describe B4010 conformation, a previous study has demonstrated that it can consistently predict conformations of branched antimicrobial peptide B2088 in both aqueous and lipid environments [34]. For the lipids, the recently released CHARMM36 force field was used because it predicts reasonable membrane properties in a tensionless ensemble [35]. Initially, the peptide was placed close to the fungal membrane with the initial conformation taken from the end of a 100 ns simulation in pure water. The peptide-membrane complex was solvated with TIP3P water and counter ions were added to neutralize the system. Before the production stage of the MD simulation, the system was subjected to 500 steps of energy minimization using the steep descent algorithm, followed by 100 ps of NVT simulation, which was followed by an MD simulation of 400 ns using the GROMACS 4.5 package [36]. During the MD simulations, both LJ and real-space electrostatic interactions were treated using a cutoff potential at a distance of 1.2 nm, while the long-range electrostatic interactions in reciprocal space were calculated using the particle-mesh Ewald algorithm [37]. The covalent bonds involving hydrogen atoms were constrained using the LINCS algorithm which allowed us to use a time step of 2 fs in all the simulations [38]. The simulations were run in the NPT ensemble with the temperature coupled to an external heat bath at 310 K through the Nose-Hoover method and pressure maintained at 1 atm using the Parrinello-Rahman method with semi-isotropic coupling [39]. The electrostatic potential maps for peptide adsorbed or peptide-free bilayer were calculated as previously reported [40].

### Scanning Electron Microscopy

*Candida alibicans* (ATCC 10231) was incubated with 20µg/mL of B4010 (MIC) at 370C and 200 µl of a fungal suspension was removed after 30 minutes. The suspension was centrifuged at 2000 rpm for 2 minutes; the pellet was washed twice with phosphate buffer, prefixed in 0.5 ml of a mixed aldehyde fixative (2% glutaraldehyde and 2% paraformaldehyde in 0.1 M sodium cacodylate buffer) for 24 hours. Then the pellet was washed once again in sodium cacodylate buffer (Electron Microscopy Sciences, Washington, USA) and the fungal cells suspension was subsequently mounted on poly-L-Lysine coated cover-slips and post-fixed in 1% osmium tetroxide (Electron Microscopy Sciences). Following dehydration in a graded series of ethanol, the samples were critical-point-dried and sputter coated with 10 nm of gold. All samples were viewed and photographed on a FE SEM (Supra 55 VP - Zeiss) with an accelerating voltage of 3 kv at Carl Zeiss facility, National University of Singapore. Candida incubated in PBS was considered as positive control and processed in the same way.

## Results

### Antifungal Properties of Linear and Multivalent Peptides

**Figure 1:**
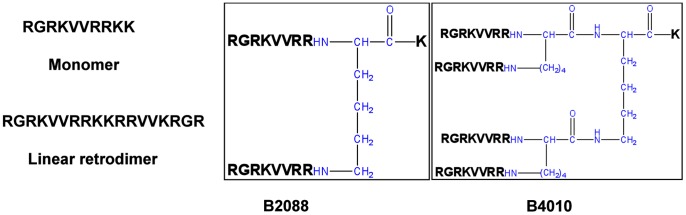
Schematic representation of the peptides used in this study. The branched lysine is colored in blue. For the scrambled peptide (Sc_B4010) each copy contains the sequence VRGRVRKR.

The peptide RGRKVVRRKK displayed weak antifungal activity (***Table 1***) against *C. albicans* strains. However, upon linking two copies of the sequence through a branched lysine (B2088, **Figure 1**), substantial decrease in MIC values were observed as compared to the monomer or linear retrodimer (RGRKVVRRKKRRVVKRGR) peptides (***Table 1***). Further assembling the two copies of branched dimer through branched lysines produced a tetrabranched peptide (B4010) which decreased the MIC by an additional 4–10 fold compared to the co-valent dimer (***Table 1***). The MIC values of B4010 (0.37 µM) for two clinical isolates of *C. albicans* were also lower when compared to the MIC values for amphotericin B (1.4 µM) and natamycin (15 µM), the latter is the only US FDA approved antifungal for ophthalmic applications [41]. Interestingly, scrambling the sequence of B4010 (Sc_B4010) led to 2–4-fold increase in MIC values, yet retained significant potency.

**Table 1:** MIC of synthetic linear and branched peptides against various yeasts and fungi.

*Strains of Yeasts/Fungi*	*MIC in µM of*					
	Monomer	Linear Retrodimer	B2088	B4010	Sc_B4010	*Natamycin*
*C. albicans* ATCC10231	78	22	5.5	1.4	5.6	*15*
*C. albicans* ATCC24433	n.d^a^	5.5	2.7	1.4	5.6	*15*
*C. albicans* ATCC2091	n.d	11	1.4	0.7	2.7	*7.5*
*C. albicans* DF2672R	78	2.7	0.8	0.34	0.8	*15*
*C. albicans* DF1976R	n.d.	2.7	0.8	0.34	0.8	*15*
*F. solani* ATCC36031	>78	n.d.	10.9	1.4	n.d.	*4.7*
*F. solani* DM3782	n.d.	n.d.	n.d.	1.4	n.d.	*2.4*
*F. solani DF1500*	*n.d.*	*n.d.*	*n.d.*	*1.4*	*n.d.*	*2.4*

a Not determined.

**Figure 2:**
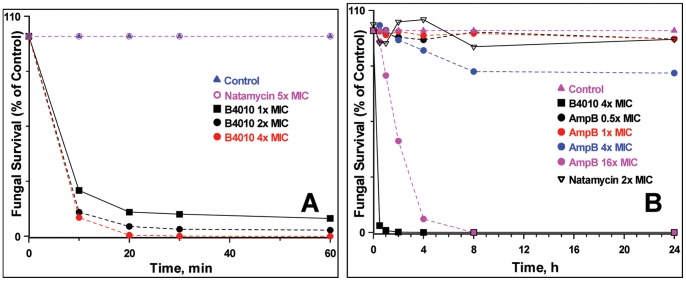
Time kill curves for B4010 against *C. albicans*. (A) ATCC 10231 and (B) clinical isolate DF2672R. The yeasts cells were incubated with various concentrations of peptide or antifungals in SD broth. At the indicated times, survivors were diluted and plated to allow colony counts.

CD spectra displayed strong negative minima around 200 nm for both linear as well as branched dimers, suggesting branched lysine did not alter the secondary structure. Similarly no discernable differences were observed for B4010 and Sc_B4010 tetrabranched peptides, suggesting that scrambling the sequence did not influence the secondary structure (**Figure *S1***). Concentration-dependent time-kill experiments were conducted after exposing *C. albicans* to B4010 and polyene antifungals (**Figure 2A and 2B**). For the ATCC strains, B4010 induced ∼91% killing in 1 h at 1× MIC whereas ∼97% killing was observed as the concentration was increased to 2× MIC. Increasing the concentration of B4010 to 4× and 8× MIC values led to 4 log reduction (99.99%) in viable *Candida* cells within 20 minutes and 10 minutes, respectively. In contrast, natamycin, even at 16× MIC, required 24 h to achieve similar endpoints. We have also compared the kill-kinetics of B4010, natamycin and amphotericin B against the clinical isolates *C. albicans DF2672R* strains. At 4× MIC, B4010 elicited complete killing in 1 h whereas for amphotericin B maximal effect was achieved in less than 8 h at 16× MIC. However, no fungicidal action was observed for natamycin at 2× MIC even after 24 h exposure.

**Figure 3:**
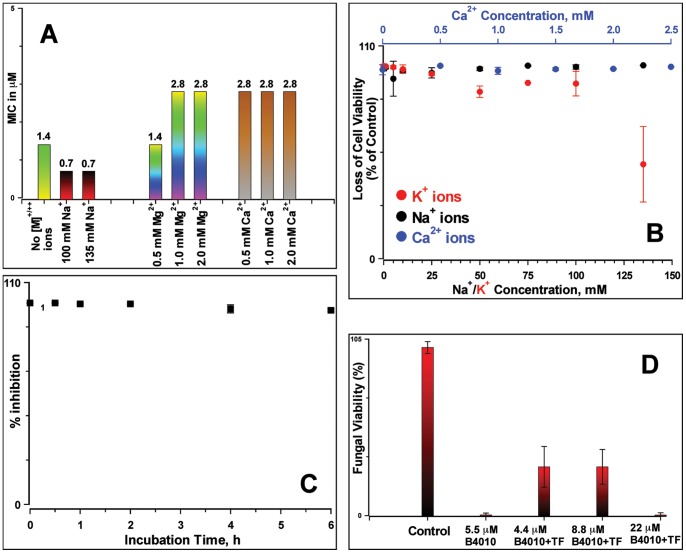
Antifungal properties of B4010 in the presence of metal ions and complex biological fluids. (A) MIC values determined in the presence of monovalent and divalent metal ions. The numbers above the bars indicate the determined MIC values. (B) Candidacidal properties of B4010 in the presence of metal ions. The concentration of B4010 was 5.5 µM. (C) Antifungal activity in the presence of trypsin. (D) Candidacidal activity of B4010 in the presence of 50% rabbit tear fluid.

Next, we examined the effects of physiological concentrations of metal ions (NaCl, CaCl_2_ and MgCl_2_) on the antifungal activity of B4010. The presence of 135 mM NaCl amiolerated the antifungal activity, decreasing the MIC by 2-fold whereas physiological concentrations of divalent cations resulted in only a 2-fold increase in MIC values (**Figure 3A**). However, addition of 100 mM KCl resulted in an increase in MIC by a factor >16 compared to the medium not supplemented with KCl. We have also determined the cation sensitivity on the candidacidal activity of B4010. Addition of B4010 (at 5.5 µM) resulted in a complete loss of viable cells in the presence of monovalent and divalent metal ions (**Figure 3B**). However, in the presence of a high concentration of KCl, slight decrease in the candidacidal activity was observed.

### Antifungal Activity of B4010 in Trypsin, Tear Fluid and Serum

Antifungal properties of the peptide were examined in three complex biological environments. The peptide was incubated with trypsin (trypsin:peptide ratio = 1∶100) at 37°C. An aliquot of this mixture was withdrawn at various time intervals, added to the *C. albicans* inoculum and the growth was monitored (**Figure 3C**). No loss of antifungal activity was found up to 6 h incubation with trypsin, suggesting improved proteolytic resistance of tetrabranched peptides. Mass spectrometry analysis indicated significant presence (∼20%) of intact B4010 after 6 h incubation with trypsin (***Figure S2***).

The antifungal efficacy of B4010 was also monitored in the presence of complex biological fluids such as tear fluid and human serum. In the absence of tear fluid, about 98±2% loss of viable cells were observed at a peptide concentration of 5.5 µM. However, the candidacidal activity was moderately suppressed by the presence of 50% tear fluid at lower concentration (**Figure 3D**). At 22 µM, however, no loss of activity was observed suggesting a higher concentration of peptide was needed to achieve more than 3 log reduction of viable *C. albicans* in the presence of tear fluid. The effect of serum on antifungal efficacy of B4010 was assessed by measuring the shift in the MIC of B4010 in the presence of 5% human serum against two clinical isolates of *C. albicans*. For both the strains the MIC values were shifted from 0.37 µM without serum to 5.5 µM in the presence of serum, suggesting 16-fold increase in the MICs in 5% sera.

### Antifungal Activity of B4010 against *ergΔ* Strains

The emergence of resistant pathogens poses a greater threat to the management of fungal infections [5]–[7]. To determine if the sterol alterations have an affect on the antifungal activity of B4010, experiments were performed using *S. cerevisiae* strains that carry specific mutations in the ergosterol (*ergΔ*) pathways. **Table 2** summarizes the MIC values of B4010 against wild type and various *ergΔ* mutants which carry altered sterol structure and compositions. Interestingly, all the *ergΔ* mutants displayed hyper susceptibility to B4010 i.e. 2–4 fold decrease in MIC values compared to the wild type strains.

**Table 2:** MIC of B4010 against *S. cerevisiae* mutants carrying altered sterol structure and composition.

Yeast Strains	Relevant genotype	Sterol Content (%)^a^	MIC, µM
RH448	Wild type	Ergosterol (77%)	*5.5*
RH5812	*erg2Δ*	Ergosta-8, 24(28)-dienol (24.5%), Ergosta-8-enol (23.8%)	*1.4*
RH4213	*erg3Δ*	Ergosta-7,22-dienol (45.9%)	*1.4*
RH5930	*erg3Δerg6Δ*	Zymosterol (40%), Cholesta-7,24-dienol (39.5%)	*1.4*
RH5873	*erg4Δ erg5Δ*	Ergosta-5,7,24-trienol (72.2%)	*1.4*
RH3616	*erg2Δerg6Δ*	Zymosterol (85.6%)	*2.8*
*RH5684*	*erg6Δ*	Zymosterol (39.4%), Cholesta-5,7,24-trienol (32.3%)	*2.8*

a Taken from [29].

### Effect of B4010 on Haemolysis, Cytotoxicity, Corneal Reepithelialization Rate and *in vivo* Toxicity

**Figure 4:**
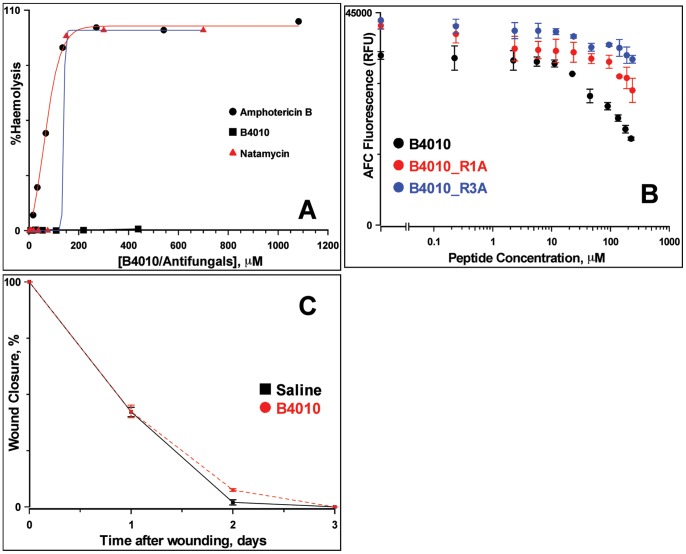
Toxicity and corneal reepithelialization of B4010. (A) Haemolytic activity of B4010 and other antifungals against 4% rabbit erythrocytes. (B) Cytotoxicity of B4010 and two alanine substituted B4010 to HCE cells. (C) Effect of B4010 on corneal reepithelialization of New Zealand white rabbits as measured by fluorescein staining. The peptide concentration was 22 µM.

The ability of B4010 in disrupting the membrane integrity of mammalian cells was evaluated by haemolytic assay using rabbit red blood cells. The peptide had no significant haemolytic activity (<1% haemolysis) to rabbit erythrocytes even at 440 µM (**Figure 4A**). On the other hand amphotericin B and natamycin displayed significant haemolytic activity at low concentration and the HC_50_ values were 59.4±7.8 µM and 139.6±0.18 µM, respectively. Cytotoxicity was assessed after 24 h exposure of human conjunctival epithelial cell lines to the peptides/drugs and the EC_50_ (effective concentration of peptide that decreased the viable cells by 50%) was determined. Exposure of B4010 caused 50% loss of cell viability at 220 µM (**Figure 4B**). The EC_50_ value of B4010 was comparable to natamycin (211.7±20.5 µM) and better than amphotericin B (134±16 µM, ***Figure S3***). We have further examined whether topical application of B4010 influences the corneal wound healing *in vivo*. The epithelial wound healing rate was not altered by topical application of B4010 (22 µM) 3 times a day compared to the control groups (**Figure 4C** **and** ***Figure S4***). This is an important result as corneal epithelial wound healing re-establishes a critical component of innate immunity for the eye. Preliminary *in vivo* toxicity tests on mice indicated no sign of mortality, morbidity or toxicity when injected by IP (200 mg/kg) or IV (100 mg/kg) routes.

### Membrane Disrupting Activity of B4010

**Figure 5:**
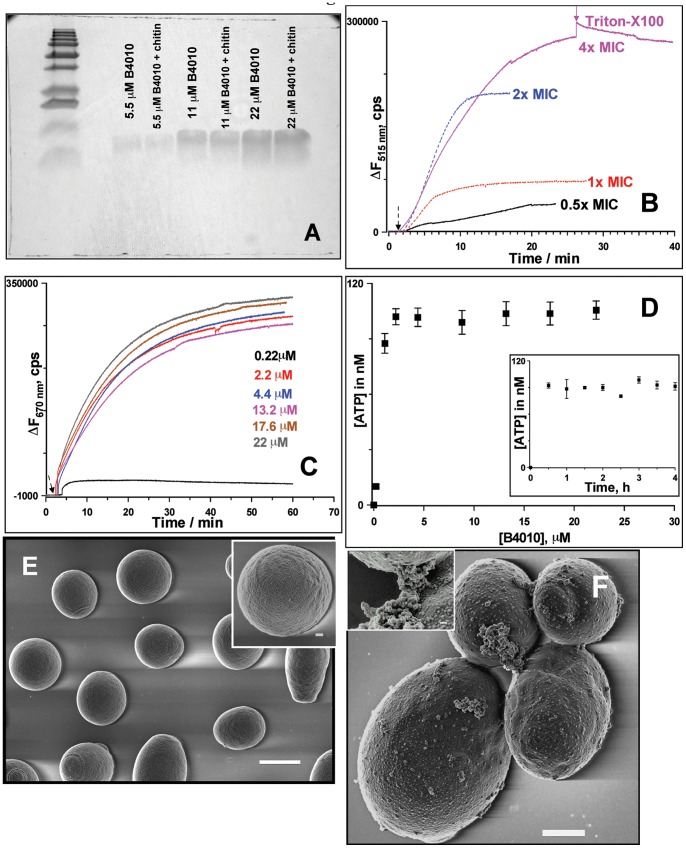
Effect of B4010 on cytoplasmic membrane potential, membrane permeabilization and morphology of *C. albicans*. (A) SDS-PAGE showing lack of affinity of B4010 for insoluble chitin. (B) SYTOX Green uptake of *C. albicans* induced by varying concentration of B4010. (C) B4010-mediated membrane depolarization monitored by diSC_3_5 assay. (D) B4010-induced extracellular ATP release of *C. albicans*. The inset shows the kinetics of ATP release. B4010 concentration was 5.5 µM. (E) SEM of untreated *C. albicans*. Scale bar is 2 µm (inset scale bar = 200 nm). (F) SEM image of C. albicans treated with 5.5 µM B4010. Scale bar is 1 µm (inset scale bar = 100 nm).

To probe the mechanism of action, we first examined the affinity of B4010 for insoluble polysaccharides such as chitin and β-D-glucan, the major components of fungal cell walls. The peptide showed no affinity for cell wall polysaccharides as no co-precipitation was observed with either chitin or β-D-glucan (**Figure 5A**). Therefore, two complementary fluorometry assays were performed to determine if B4010 had a membrane targeting action, a characteristic feature of cationic antimicrobial peptides. To check if the peptide permeabilized the cytoplasmic membrane, we assayed uptake of SYTOX Green dye. This is a membrane impermeable dye and does not fluoresce when incubated with cells which have intact cell membrane. However, in membrane-compromised cells, the dye fluoresces strongly upon binding to intracellular nucleic acids [42]. Addition of B4010 to *C. albicans* incubated with 1 µM SG resulted in rapid uptake of the dye with a concomitant increase in fluorescence intensity in a concentration-dependent manner (**Figure 5B**). At ½× MIC (13%) and 1× MIC values (24%), a weak permeation was observed. About 70% cells were membrane compromised after the addition of B4010 at 2× MIC in 10 mins. However, a higher level of dye uptake (>90%) was observed within 25 mins when the concentration of B4010 was increased to 4× MIC.

Since yeasts maintain a negative resting membrane potential inside the cell, we determined if the candidacidal action of B4010 was due to dissipation of electrochemical gradients across the membrane, using a potential sensitive probe, diS-C_3_-5. **Figure 5C** shows the concentration-dependent dissipation of membrane potential upon addition of B4010 using *C. albicans* loaded with the potential sensitive probe. The time course of depolarization occurred instantaneously upon addition of B4010, indicating release of probe from the cells. However, addition of peptide 2× above the MIC had no significant influence on the final membrane potential dissipation. To verify if the dissipation of membrane potential is linked to candidacidal activity, we have also performed the fungal viability assay under identical conditions without the probe. A complete loss of viability was observed when the concentration of peptide exceeded 4× MIC (data not shown). Together with the SG uptake assay, these results confirmed that B4010 caused rapid membrane potential dissipation and permeabilizes the cytoplasmic membrane of *C. albicans* in a concentration-dependent manner.

As the perturbation of membrane would affect its barrier function and accompanied by release of essential intracellular components, we have assessed the extracellular release of metal ions and ATP upon challenging *C. albicans* with B4010. The background K^+^ and Ca^2+^ concentration in the supernatant were 43.4±2 µM and 2.5±0.5 µM, respectively. After incubation of *C. albicans* with B4010 (5.5 µM) for 2 h, more than two fold elevation of potassium (104±4.2 µM) and calcium (5.8±1.3 µM) concentration was observed, whereas no significant changes in the levels of Na^+^ and Mg^2+^ ions were observed. The release of ATP was dependent on the concentration of peptide with a maximum ATP release was achieved above 4× MIC of B4010 (**Figure 5D**). At 4× MIC, ATP bioluminescence assay further indicated a rapid release of ATP (∼30 minutes) from *C. albicans* upon addition of B4010 (**Figure 5D** **inset**).

Morphological changes in *C. albicans* treated with B4010 were investigated by scanning electron microscopy. Untreated cells displayed round, dome-shaped morphologies with a smooth surface (**Figure 5E**). However, the cell surface of *C. albicans* incubated with B4010 for 30 mins revealed severe damage with bud scars appearing on the surface (**Figure 5F**). In certain cells release of irregular material was also visible (**Figure 5F** *inset*), suggesting membrane-lytic action of B4010.

### Effects of Proton Uncouplers and Metabolic Inhibitors on the Antifungal Activity of B4010

**Figure 6:**
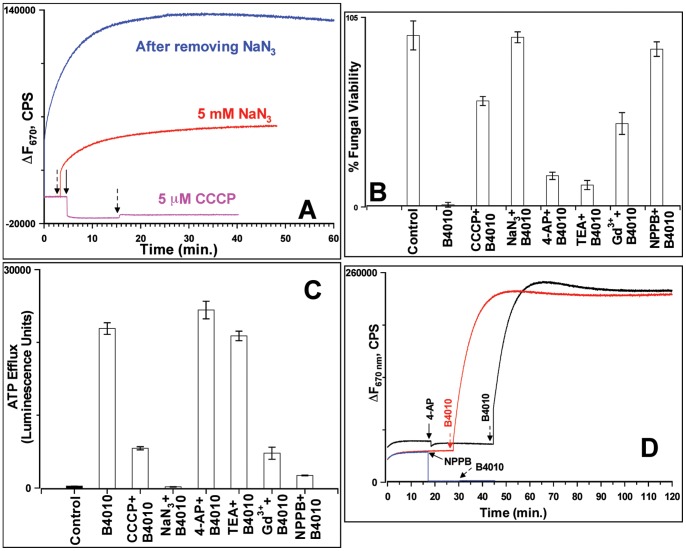
Effects of various additives on candidacidal and membrane permeabilizing properties of B4010. (A) Effect of CCCP and NaN_3_ on membrane potential. (B) Effect of various additives on (B) viability and (C) ATP release. (D) Effect of ion-channel inhibitors on membrane potential. The colored arrows indicate the time of addition of additives whereas the black arrows indicate B4010.

To study the effect of metabolic activity of *C. albicans* on the susceptibility to B4010, the diS-C_3_-5 loaded cells were incubated with 5 µM CCCP (an uncoupler of proton gradients) or 5 mM NaN_3_ (which blocks both classical and alternative pathways of mitochondrial inhibition) and monitored the changes in fluorescence intensity upon addition of B4010. Addition of CCCP resulted in a strong reduction in fluorescence intensity indicating collapse of the membrane potential. Subsequent addition of B4010 had weak changes in the transmembrane potential (**Figure 6A**). As shown before by Veerman et al., addition of NaN_3_ to dye-loaded cells caused weak depolarization [43]. Subsequent addition of B4010 resulted in an increase in fluorescence intensity (**Figure 6A**). However, ∼25 fold reduction in fluorescence intensity was observed in the azide-treated cells compared to control cells. Removal of NaN_3_ by centrifugation reversed the intensity change, indicating that the energy poison inhibits the candidacidal activity reversibly. These results suggested that both proton uncoupler and energy poison significantly decreased the B4010-induced depolarization of the cytoplasmic membrane.

The dependence of candidacidal activity on the energy metabolism was further assessed by fungal viability and extra cellular ATP release assays. B4010 (5.5 µM) added to *C. albicans* resulted in 98±2% loss of viable cells (**Figure 6B**). However, addition of B4010 to cells preincubated with 5 µM CCCP or 5 mM NaN_3_ resulted in only 41±2.5% or 6±2% loss of viable cells, respectively (**Figure 6B**). When tested under identical conditions without B4010, both the additives did not impair the viability of *C. albicans*. Interestingly, the loss of antifungal activity of B4010 in the presence of NaN_3_ is reversed by benzyl alcohol. Addition of B4010 to *C. albicans* pretreated with NaN_3_ and 60 mM benzyl alcohol resulted in significant loss of viability (85±7%).

The effect of CCCP/NaN_3_ on ATP release by B4010 was also investigated. Consistent with the cell viability assays, ATP bioluminescence assay indicated significant increase in the extracellular ATP levels in B4010-treated cells (**Figure 6C**). However, cells pretreated with CCCP/NaN_3_ followed by the addition of B4010 resulted in profound reduction in ATP release compared to B4010 treated cells (**Figure 6C**).

### Effects of Ion-channel Inhibitors on Antifungal Activity of B4010

Our observations that B4010 caused rapid release of K^+^ as well as ATP and that the addition of external K^+^ decreased the extent of cell death with >16-fold increase in MIC at elevated ion concentrations prompted the question whether ion-channel inhibitors could protect *C. albicans* from B4010. We have tested effects of non-specific organic cationic ion-channel inhibitors (TEA and 4-AP) as well as yeast stretch-activated ion channel blocker Gd^3+^ on candidacidal activity of B4010. The peptide caused significant loss of viability in cells that were pretreated with TEA and 4-AP (**Figure 6B**). Consistent with the loss of viability, a similar amount of ATP was released from the cells pretreated with TEA and 4-AP after B4010 addition as was observed without these inhibitors (**Figure 6C**). On the other hand, cells incubated with Gd^3+^ provided partial protection (54±6% loss of viability) against B4010-induced killing. Addition of B4010 to *C. albicans* cells pretreated with the anion channel inhibitor, NPPB, conferred significant protection (12±4% loss of viability) of *C. albicans* from B4010 (**Figure 6B**). ATP release assays further confirmed the absence or reduced ATP release from additives that conferred complete or partial protection (**Figure 6C**).

To determine if the loss of B4010 activity in the presence of NPPB was associated with alterations in the membrane potential, we performed diS-C_3_-5 assay in the presence of additives followed by the addition of B4010. TEA or 4-AP did not alter the membrane potential whereas subsequent addition of B4010 resulted in complete loss of membrane potential and the magnitude of intensity changes was similar to the one observed in cells without prior addition to 4-AP or TEA (**Figure 6D**). When Gd^3+^ was added to dye-loaded *C. albicans*, a significant depolarization was observed. Further addition of B4010 caused weak dissipation of membrane potential, augmenting the fungal viability and ATP release assays (data not shown). A similar effect was observed when 100 mM KCl was used (***Figure S5***). *C. albicans* incubated with NPPB resulted in collapse of membrane potential without affecting the fungal viability. Further addition B4010 to NPPB-treated cells prevented dissipation of membrane potential (**Figure 6D**) and abrogated the candidacidal properties of B4010. The dye release assays in the presence of various additives suggested that a resting negative membrane potential and metabolic activity are crucial for candidacidal activity of B4010.

### B4010 Induced Calcein Leakage from Phospholipid Vesicles

**Figure 7:**
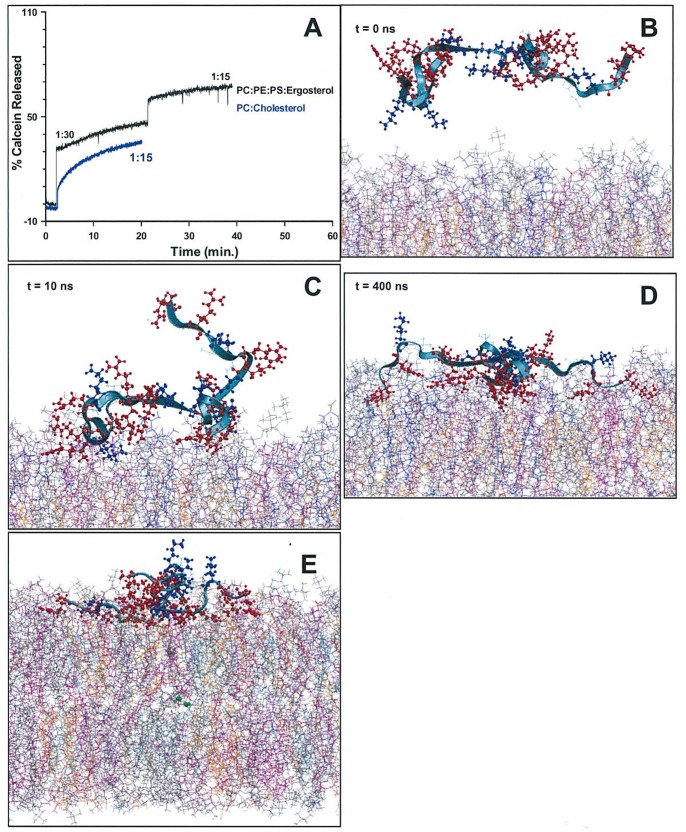
Interaction of B4010 with model membrane. (A) Time course of calcein release from SUVs of PC:PE:PS:erg and PC:cholesterol. The peptide:lipid ratio is indicated in the graphs. (B)-(D) Snapshots illustrating the interaction between B4010 and mixed bilayer containing ergosterol. The acyl chains of the aggregated POPC (grey), POPE (cyan), POPS (pink) and ergosterol (orange) are presented in line form. The peptide backbone is shown in ribbon form. (E) Translocation of water molecules (green) from inner leaflet to the outer as a consequence of membrane perturbations caused by B4010.

The ability of B4010 to induce membrane perturbation in phospholipid vesicles that did not contain proteins was investigated by calcein release from SUVs containing various lipid compositions. Two lipid mixtures, PC containing cholesterol and PC:PE:PI/PS containing various amounts of ergosterol were used to study the ability of B4010 to induce membrane lysis. About 60–70% calcein-release was observed from PC:PE:PI SUVs containing various % of ergosterol (***Figure S6A***). Thus, for further studies, we used PC:PE:PS SUVs containing 15% ergosterol. At peptide:lipid ratio of 1∶30, B4010 induced ∼46% dye release which increased to ∼68% when the peptide concentration was doubled (**Figure 7A**). For B2088 and Sc_4010, under identical conditions, a decreased calcein release was observed (***Figure S6B***). On a zwitterionic SUVs containing PC and cholesterol B4010 caused ∼36% calcein release.

### Interactions of B4010 with Lipid Bilayer Investigated by MD Simulations

To obtain atomistic details of lipid binding and membrane penetration properties of B4010, we have combined biophysical results with molecular dynamics simulations. Simulating the interaction of B4010 with model membranes which mimic the composition of microbial and mammalian cytoplasmic membrane provides useful information such as the selectivity of the peptides and key residues involved in the peptide-lipid interactions [44], [45]. Due to the difficulties in obtaining structural details by NMR spectroscopy (arising from unstructured nature of B4010 in buffers as well as in model SUVs), we used MD simulations to gain atomistic information on B4010-membrane interactions. When placed at a distance of ∼30 Å away from the bilayer surface B4010 remained in an extended conformation with a radius of gyration of 1.24 nm (**Figure 7B**). Simulation studies revealed that B4010 diffused rapidly (within ∼1 ns) from aqueous solution into the water-bilayer interface, and adsorbed stably onto the mixed bilayer at around 30 ns and remained stable even at longer simulation times (***Figure S6C***). At t = 10 ns, two branches of the peptide stably attached to the bilayer while the other two branches remained in the aqueous phase (**Figure 7C**). As the simulation time increases, all four branches adsorbed and spread on the membrane surface adopting an extended conformation (**Figure 7D**). With increasing simulation time, the structure of the peptide gradually stabilized with an increasing number of favourable hydrogen bonds between basic residues of the peptide and lipid acceptor groups. During the simulation, translocation of water molecules across the membrane was observed, suggesting that the peptide caused considerable perturbation and enhanced membrane permeability (**Figure 7E**).

**Figure 8:**
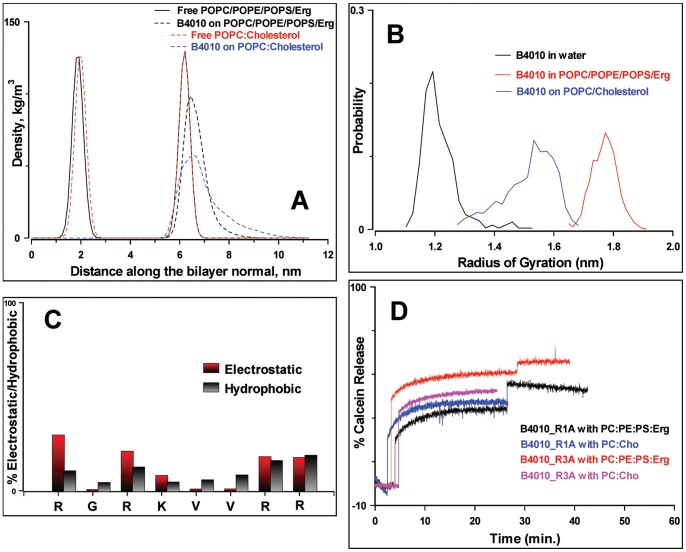
Interaction of B4010 and other peptides with model membrane. (A) Density distribution functions of B4010 along the bilayers of two model membrane. The distribution of phosphorous atoms of the bilayer is shown for a reference. (B) Distribution of radius of gyration of B4010 in water and in two model bilayer systems. (C) Average residue-wise contribution to electrostatic and hydrophobic interactions of B4010 to mixed bilayer containing ergosterol. (D) Time course of calcein release from alanine substituted B4010 in two different SUVs.

However, the peptide displayed weak affinity for the bilayer containing POPC and cholesterol. The calculated average distances of the peptide relative to the average phosphate positions in the lipid leaflets showed slower adsorption kinetics compared to mixed bilayers containing ergosterol (***Figure S6C***). **Figure 8A** shows the density profiles of B4010 during the final stages of the simulations in both the bilayers. The peptide inserted more deeply into the mixed bilayer containing ergosterol than in the POPC/cholesterol mixture. To analyze the compactness, we have estimated the radius of gyration of peptide in water and in lipids (**Figure 8B**). The radius of gyration of B4010 in water was 1.32±0.13 nm. Upon interaction with the mixed bilayer containing ergosterol, the peak of distribution was shifted to larger values of 1.8±0.08 nm. However, in the presence of POPC/cholesterol bilayer a broad distribution with a maximum around 1.48±0.13 nm was observed (**Figure 8B**). CD studies confirmed that the peptide remained in an extended conformation in the presence of SUV containing mixed liposome with no discernable structural transitions to α-helical or β-sheet structure was observed, thus supporting the simulations results (***Figure S6D***). We calculated the 2D-density distribution of lipids and ergosterol around the peptide in order to analyze the preference of B4010. POPS lipid displayed the highest density around the peptide followed by POPE and POPC lipids (***Figure S7***). It was observed that the POPS molecules preferentially interacted with the peptide, while the ergosterol molecules were excluded from the peptide, resulting in some POPS-rich and some ergosterol-rich domains (***Figure S7***).

To validate the MD simulation results that B4010 had no or weak interactions with membrane bound ergosterol we have performed isothermal titration calorimetry (ITC) studies. The measurements were performed by titrating SUV of PC:ergosterol to the peptide solution in the sample cell. The results confirmed absence of any heat changes upon addition of PC:ergosterol SUVs to peptide (***Figure S8A***). However, a substantial increase in the net exotherm was observed when the SUV was titrated against the polyene antifungals amphotericin B or natamycin (***Figure S8B and Figure S8C***). These results indicate strong affinity of the polyenes and lack of affinity of B4010 for membrane bound ergosterol.

### Identification of Amino Acids Residues Critical for Interaction with Model Lipids

MD simulations indicated that when B4010 binds to the mixed bilyaer, it causes redistribution of phosphate groups in the upper leaflet. To rationalize the membrane-disruptive action of B4010, it was necessary to identify the critical amino acids that selectively disrupt the mixed bilayer. **Figure 8C** shows the average % electrostatic interactions for the mixed bilayer system in terms of position of the amino acid residues in the 4 copies. It should be noted that the calculations take into account only the enthalpic contributions from peptide-lipid interactions although the binding is driven by both entropic as well as enthalpic contribution form peptide-water interactions. Nevertheless, **Figure 8C** describes qualitatively the enthalpic contributions of each amino acid to the electrostatic interactions. The analyses suggested that the first arginine residues of the 4 copies in B4010 participate more strongly with the mixed lipids compared to the other arginine or lysine residues in the sequence contributing ∼30% of total electrostatics.

**Figure 9:**
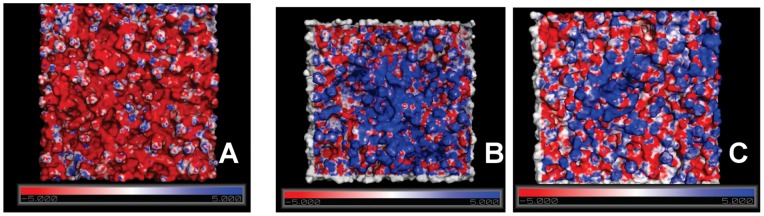
Electrostatic potential map on the adsorption of B4010 and B4010_R1A with bilayer. (A) POPC/POPE/POPS/Erg bilayer (B) B4010-adsorbed bilayer and (C) B4010_R1A-adsorbed bilayer. The negative and positive surfaces are labeled in red and blue, respectively whereas grey color indicates neutral surface.

To verify the importance of this residue, we replaced the first arginine residue with alanine (B4010_R1A) and determined the % calcein released, MIC against 5 different *C. albicans* strains and cytoxicity of the peptide. The modified peptide caused 48% calcein leakage from PC/PE/PS/Erg and 42% dye release from PC/cholesterol SUVs and caused 2–4× increase in MIC values compared to B4010 (**Figure 8D** and ***Table 3***). Cytotoxicity experiments on HCE cells indicated that at 235 µM, the peptide caused 32.3% cell death compared to 40.1% observed for B4010. To understand the difference in antifungal and membrane-lytic activities, we performed 400 ns simulations of the peptide B4010_R1A. **Figure 9** depicts the electrostatic map of B4010 and B4010_R1A adsorbed on to the model membrane containing ergosterol. Though, both peptides displayed strong adsorption onto the membrane, B4010 elicited significant changes in the electrostatic potentials compared to B4010_R1A.

**Table 3:** MIC, cytotoxicity and calcein release properties of tetravalent peptides.

Peptides/antifungals	MIC in µM against					EC_50_, µM^a^	% Calcein released from	
	Ca 10231	Ca 24433	Ca 2091	Ca 2672R	Ca DF1976R		PC/PE/PS/Erg^c^	PC/Cho
**B4010**	1.4	1.4	0.7	0.37	0.37	220	68	35.3
**B4010_R1A**	5.9	5.9	1.5	1.5	0.75	>237 (67.6)^b^	42	41.7
**B4010_R3A**	1.5	1.5	1.5	0.4	0.4	>237 (80.9)^b^	63	48
**Amphotericin B**	1.35	1.35	<0.4	1.35	1.35	139.5±19.6	0	n.d.
**Natamycin**	15	15	7.5	15	15	211.7±20.5	0	n.d.

a Effective concentration required to kill 50% of human conjunctival epithelial cells.

b Values in parenthesis indicate cell viability in % at the maximum concentration tested.

c % calcein released at peptide: liposome ratio 1∶15. n.d. is not determined.

To confirm that the reduction in antifungal activity of B4010_R1A was not caused by decrease of charge but due to specific interaction of the first arginine residue with cytoplasmic membrane, we replaced the 3^rd^ residue with alanine and compared the membrane lytic and antifungal properties. Replacement of the internal arginine (B4010_R3A) caused ∼63% calcein release from PC/PE/PS/Erg SUVs (**Figure 8D** and ***Table 3***). Except for one *C. albicans* strains, the MIC was not significantly altered for this peptide compared to B4010 (***Table 3***). Compared to B4010, R3A peptide displayed less cytotoxicity to HCE cells.

## Discussion

In tropical countries, mycoses have high incidence and are responsible for major health and economic problems [46]. The expanding populations of immuno compromised patients, large-scale use of antifungals in food and increased use of medical devices and implants have further raised the incidence of fungal infections. Systemic candida species account for 40% mortality rates and is the 4th leading cause of hospital acquired blood stream infections in the US [47], [48]. Due to increase in antifungal resistance and limited choice of antifungals, AMPs that interfere with membrane physiology and in many cases have multiple targets within cells, are attractive drug candidates [8], [9]. In this study, we report that multimerization of a weak antifungal peptide resulted in highly enhanced antifungal and membrane permeabilizing activities compared to its monomer. A comparison between branched and linear retrodimer suggested that branching through lysine core resulted in 2–4× decrease in the MIC values. Increasing the number of putative copies further decreased the MIC values. The most active peptide which carried 4 copies (B4010) displayed excellent antifungal activities against *Candida* and *Fusarium* strains. It should be noted that the tetrabranched peptide displayed better antifungal activity compared to azoles or polyene antifungals. Scrambling the sequence resulted in no apparent changes in secondary structure but 2–4 fold decrease in antifungal activity, thus indicating the importance of amino acid sequence.

Kinetics of candidacidal activity for B4010 was compared with polyene antifungals. For both the ATCC and clinical isolates, B4010 caused 3 log reductions of viable cells in less than l h at 2× or 4× MIC values whereas the polyene antifungals require an elevated concentration and longer time to achieve similar end points. Since ergosterol is the principal target of polyene antifungals, these results highlighted rapid and potent candidacidal activity of B4010. Extracellular cations antagonize the antifungal properties of AMPs and their antimicrobial potency is reduced by the lack of proteolytic stability and loss of activity in complex biological fluids [8], [11], [12], [49]. Therefore, we have evaluated the antifungal properties of B4010 in the presence of monovalent and divalent cations as well as in complex biological fluids. The results showed that B4010 retained significant antifungal activity in physiologically relevant ionic strengths although at high concentration of K^+^ ions a reduced activity was observed. The strong depolarizing activity of K^+^ on yeast cells at elevated concentration may be responsible for the partial loss of antifungal activity [50].

When incubated with trypsin or tear fluid for 6 h, B4010 retained significant antifungal potency. ESI-MS studies showed significant abundance of intact B4010 after 6 h incubation. These results suggest improved stability of B4010 in complex biological environments and augment the results reported by others that tetravalent peptides display improved proteolytic resistance compared to linear peptides [51], [52]. It is also possible that proteolysis of B4010 releases truncated peptides which still retains significant anticandida activity (and may be less potent compared to B4010) as we could detect only ∼20% of intact B4010 after 6 h incubation in trypsin. In the presence of 50% tear fluid, a 4 fold higher concentration of B4010 was required to cause complete candidacidal activity. Similarly, in the presence of human serum, about 16 fold elevation in the MIC values were observed against two clinical isolates of *C. albicans*. It is likely that the interaction of B4010 with albumin or other proteins may be responsible for the increased MIC values [29]. Taken together these results highlight considerable antifungal efficacy of B4010 in physiologically relevant milieu.

Several mechanisms have been documented in the evolution of resistance to azole and polyene antifungals by *C. albicans*. Qualitative or quantitative changes in the sterol structure and composition by altering the specific steps in the ergosterol biosynthetic pathways are amongst the most important mechanisms of azole and polyene resistance known [7]. Analysis of the sterol composition in azole-resistant *C. albicans* strains from AIDS and leukemia patients indicated the accumulation of ergosta-7,22-dienol [53]. It has been shown that mutations in *ERG4*, *ERG6* and *ERG3* of *S. cerevisiae* displayed enhanced resistance to polyene and azole antifungals [29], [54]. However, our studies showed that all the yeast mutants which carry altered sterol structure and composition are hyper sensitive to B4010. It is interesting to note that *erg3Δ* mutants that are intrinsically resistant to azoles and polyene antifungals are more susceptible to B4010 than are the erg2Δ and *erg2Δ6Δ* mutants. The enhanced membrane fluidity resulting from sterol alterations could contribute to the increased permeability of B4010, thus rendering hyper susceptibility of the mutant strains to B4010 [55].

The therapeutic potential of host defense peptides is also limited by increased cell toxicity and haemolytic activity [8]. In comparison to natamycin and amphotericin B, the peptide was non-haemolytic to rabbit erythrocytes at higher concentration. However, the cytotoxicity of the peptide to HCE cells was comparable to natamycin and superior to that of amphotericin B. B4010 did not affect the corneal reepithelialization rate in rabbit nor displayed acute toxicity in mice indicating safety of the peptide in surgical settings.

Since many antifungal peptides require an intact cell wall to exert antifungal action, we examined the affinity of B4010 for cell wall polysaccharides [56], [57]. B4010 had no affinity for β-D-glucan or chitin as no coprecipitation was observed in the pull-down experiments. To probe the membrane targeting properties and to correlate the rapid candidacidal activity, the effect of concentration of B4010 on the kinetics of membrane permeabilization was monitored by SG uptake and diS-C3-5 release assays. At all concentrations, a rapid SG uptake was observed although the maximum uptake was achieved at 4× the MIC. These results support the time-kill kinetics assays that complete killing occurred at 4× MIC in 30 minutes. diS-C3-5 dye release studies suggested a weak dissipation of the membrane potential at lower concentrations and maximum dissipation was observed above 2× MIC. A loss of viability in yeast cells exposed to 4× MIC of B4010 under identical conditions suggests that dissipation of membrane potential is linked to candidacidal activity. The rapid dissipation of membrane potential and SG uptake may indicate direct interaction of the peptide with the cytoplasmic membrane.

It has been shown that damage of the cytoplasmic membranes by peptides is accompanied by dissipation of transmembrane cation gradients and release of ATP [30]. Challenging *C. albicans* with B4010 caused 2 fold increase in K^+^- and Ca^2+^-ions indicating membrane damage. The extracellular release of ATP from yeast cells treated with B4010 is concentration-dependent with maximum efflux at 4× MIC. The kinetics of ATP efflux at 4× MIC reached a maximum with in 30 minutes, in accordance with kinetics of candidacidal activity and SG uptake. SEM studies showed that *C. albicans* treated with B4010 displayed rough with disrupted morphologies and extensive blebbing, again pointing to the observations that membrane is the principal and presumably the critical target for the peptide.

We showed that additives which alter the cytoplasmic membrane potential or energy metabolism confer substantial protection of *C. albicans* from B4010 induced lethality. At 5 µM, CCCP has been shown to cause depolarization of the cytoplasmic membrane whereas a much higher concentration (>50 µM) is required for mitochondrial depolarization [50]. Cell viability assays confirmed that cells pretreated with 5 µM CCCP provided partial protection (45% viable cells) from B4010. diS-C_3_-5 assay indicated collapse of membrane potential in the presence of 5 µM CCCP. A similar effect was observed in the presence of anion channel inhibitor, NPPB. These results suggest that the alterations in transnegative electrochemical gradient may potentially be the underlying mechanism of protection of *C. albicans* from B4010. It has been shown that the membrane potential of *S. cerevisiae* is −76±5 mV whereas for *C. albicans* the value is −120 mV [58], [59]. The reduced membrane potential of *S. cerevisiae* may account for the observed higher MIC values *S. cerevisiae* (5.5 µM) compared to *C. albicans* (0.37–1.4 µM). In support of this, a high concentration of extracellular K^+^ strongly depolarizes the yeast cells without affecting cell viability. Subsequent addition of B4010 caused little changes in the membrane potential and partial protection (40% viable cells) from B4010. Furthermore, ion-channel inhibitors, which did not alter the membrane potential, failed to protect yeast cells from B4010. However, the presence of NaN_3_ completely abrogated the candidacidal activity of B4010. It has been shown that NaN_3_ alters the fluidity of the plasma membrane of yeast without affecting the membrane potential [43]. Consistent with this, the loss of activity in the presence of NaN_3_ is restored by the addition of a membrane fluidizer (benzyl alcohol). Therefore, we suggest that the candidacidal action of B4010 is linked to plasma membrane potential and fluidity of the membrane.

The effect of B4010 on calcein-loaded SUVs containing purified lipids and ergosterol point unambiguously towards membrane-lytic action of the peptide. The results from these studies further indicated that tetrabranching and the aminoacid sequence are important for membrane disruption since a decreased calcein release was observed in the presence of B2088 and Sc_B4010 peptides. ITC studies confirmed that B4010 had weak or no interactions with PC:ergosterol SUVs, suggesting that the peptide presumably interacts with acidic lipids present in the yeasts/fungi. In cholesterol-containing zwitterionic SUVs, however, B4010 caused reduced calcein release suggesting weaker interactions with mammalian model membrane and high selectivity for the yeast/fungal model membrane. MD simulations studies revealed rapid adsorption of the peptide when placed in the presence of mixed bilayer containing ergosterol whereas interacts weakly with zwitterionic membrane containing cholesterol. A high % of calcein release and retention of extended conformation of the peptide in mixed liposome containing ergosterol supported the results obtained from the MD simulation results. In addition, the low MIC against yeasts and high EC_50_ values against HCE cells confirm that B4010 selectively damages the fungal membrane.

In the absence of structural information, MD simulations provide atomistic details of the peptide-membrane interactions. The purpose of using MD simulations is to understand the mode of interaction of peptide with mammalian and microbial model membranes and identify the critical amino acids that are responsible peptide-lipid interactions. The results help in the de novo peptide design of potent antimicrobial peptides with enhanced selectivity. Our results indicated that electrostatic interactions appear to be the dominant factor in determining the selectivity and rapid adsorption of peptide towards mixed bilayers containing ergosterol and the first arginine residue mediates the peptide-lipid interactions maximally. The decreased antifungal activity and low calcein release from model liposome for B4010_R1A peptide in which the first arginine is replaced with alanine further confirmed these observations. However, substitution of the 3rd arginine to alanine (B4010_R3A) did not affect the antifungal activity and calcein release significantly compared to B4010 indicating that reduced activity of B4010_R1A is not due to the overall net charge of the peptide and that conformational dynamics probably plays a significant role. The newly designed peptide B4010_R3A also showed higher selectivity for the fungal membrane over human, suggesting that coupling the MD simulations with experiments provide valuable insights of peptide-membrane interactions and rational design of antifungal peptides for therapeutic use.

In conclusion, we have shown that assembling 4 copies of a weakly active peptide on a branched lysine core amplifies the properties and overcomes several limitations of the linear antimicrobial peptides. In addition, the peptide is hyper potent against several yeast strains with altered sterol structure and composition, thus suggesting their potential to combat resistance. The peptide is non-toxic when tested in vitro and in vivo. B4010 likely targets the plasma membrane to cause rapid dissipation of membrane potential and loss of intracellular components. Future experimental and computer simulation studies may advance our understanding of the interaction of antifungal peptides with model lipids, and provide guidance for the rational design of therapeutically important new antifungal drugs with the promise of combating resistance.

## Supporting Information

Figure S1 CD spectropolarimetry of linear retrodimer and branched peptides in PBS (pH = 7.0). Note the absence any significant change in the secondary structure after branching. Note that all the spectra displayed characteristic CD minimum around 198–202 nm, typical of an unordered conformations.
(TIF)

Figure S2 Positive charge electrospray ionization mass spectrometry of B4010 after incubation with trypsin (enzyme:B4010 = 1∶100). The multiple charged ions are shown in red color. The various multiple charged ions that correspond to B4010 are labeled in the figure. Note the progressive decrease in the B4010 ion peaks with incubation time.
(TIF)

Figure S3 Cytotoxicity of polyene antifungals to HCE cells. The polyene antifungals were incubated with the cells for 24 h and the amount of intracellular ATP was quantified by AFC fluorescence.
(TIF)

Figure S4 Fluorescein image of the representative cornea acquired at various time points after wound. The outer broken ring in the top panels represents original wound area. The re-epithelialization rate is identical for B4010 and saline, confirming the safety of B4010 in clinical settings.
(TIF)

Figure S5 Effect of KCl on membrane potential of B4010 monitored by diSc_3_-5 fluorescence intensity at 670 nm. The blue arrow indicates time of addition of 10/100 mM KCl and the black arrows indicate time addition of B4010 (5.5 µM). Note that at higher concentration of KCl, the depolarization caused by B4010 became very weak.
(TIF)

Figure S6 (A) Calcein leakage assay from SUVs containing various % of ergosterol. In all the cases the peptide:lipid molar ratio was 1∶15. (B) Calcein release assay from mixed liposome containing 15% ergosterol in the presence of B2088 and Sc_B4010. The concentration of B2088 was doubled to match the equivalence of tetrabranched peptides. Numbers indicate the peptide lipid ratio. (C) Time course of insertion of B4010 in fungal (black) and human (red) model membranes. The values of insertion are calculated as the difference in distance between centre of mass of B4010 and the bilayer center. (D) CD sepctra of B4010 in buffer and in model lipid containing 15% ergosterol.
(TIF)

Figure S7 The 2-dimensional number density map of peptide and each type of lipids in the upper leaflet of the membrane based on the last 200 ns of a total 400 ns MD simulations. The simulations clearly show the preferential interaction between the POPS molecules and the preferential exclusion of ergosterol molecules with the peptide.
(TIF)

Figure S8 ITC heat flow traces (raw data) obtained by titrating PC SUVs containing 15% ergosterol to (A) B4010 (B) Amphotericin B and (C) Natamycin. Note that the peptide displayed very week interactions with the model lipid supporting the MD simulations results. However, the polyene antifungal amphotericin B showed pronounced heat changes upon interaction with ergosterol containing lipids.
(TIF)
