# Supplementary figures and images for: Synthetic Multivalent Antifungal Peptides Effective against Fungi
Source: PLoS One. 2014 Feb 3;9(2):e87730. doi: 10.1371/journal.pone.0087730 (PMC3912015; doi:10.1371/journal.pone.0087730)

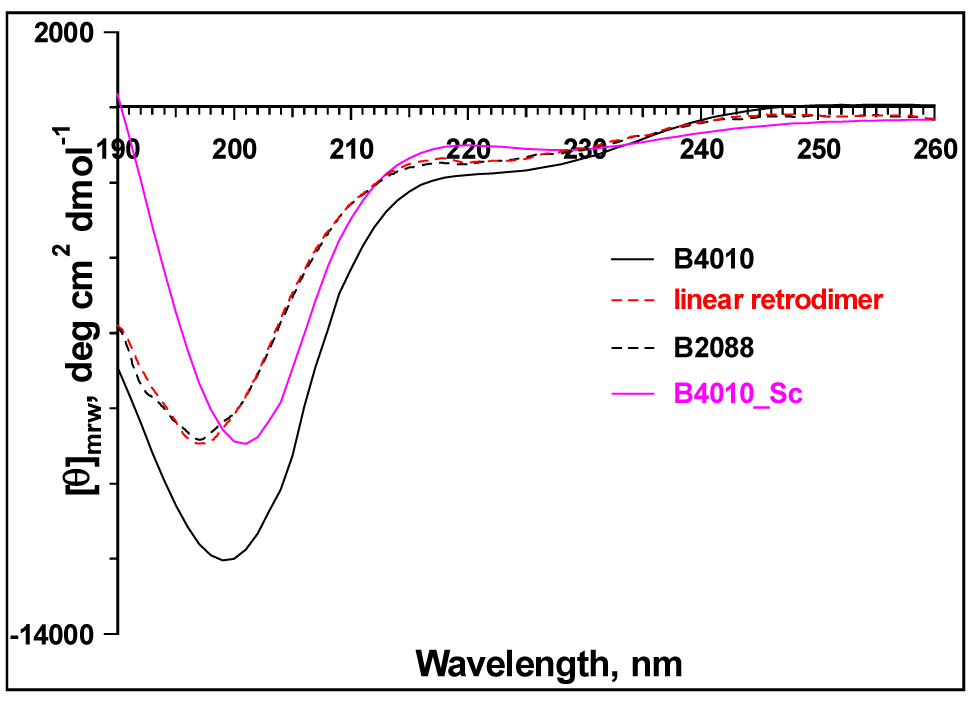

Supplement: Figure S1 — CD spectropolarimetry of linear retrodimer and branched peptides in PBS (pH = 7.0). Note the absence any significant change in the secondary structure after branching. Note that all the spectra displayed characteristic CD minimum around 198–202 nm, typical of an unordered conformations. (TIF) [file pone.0087730.s001.tif]

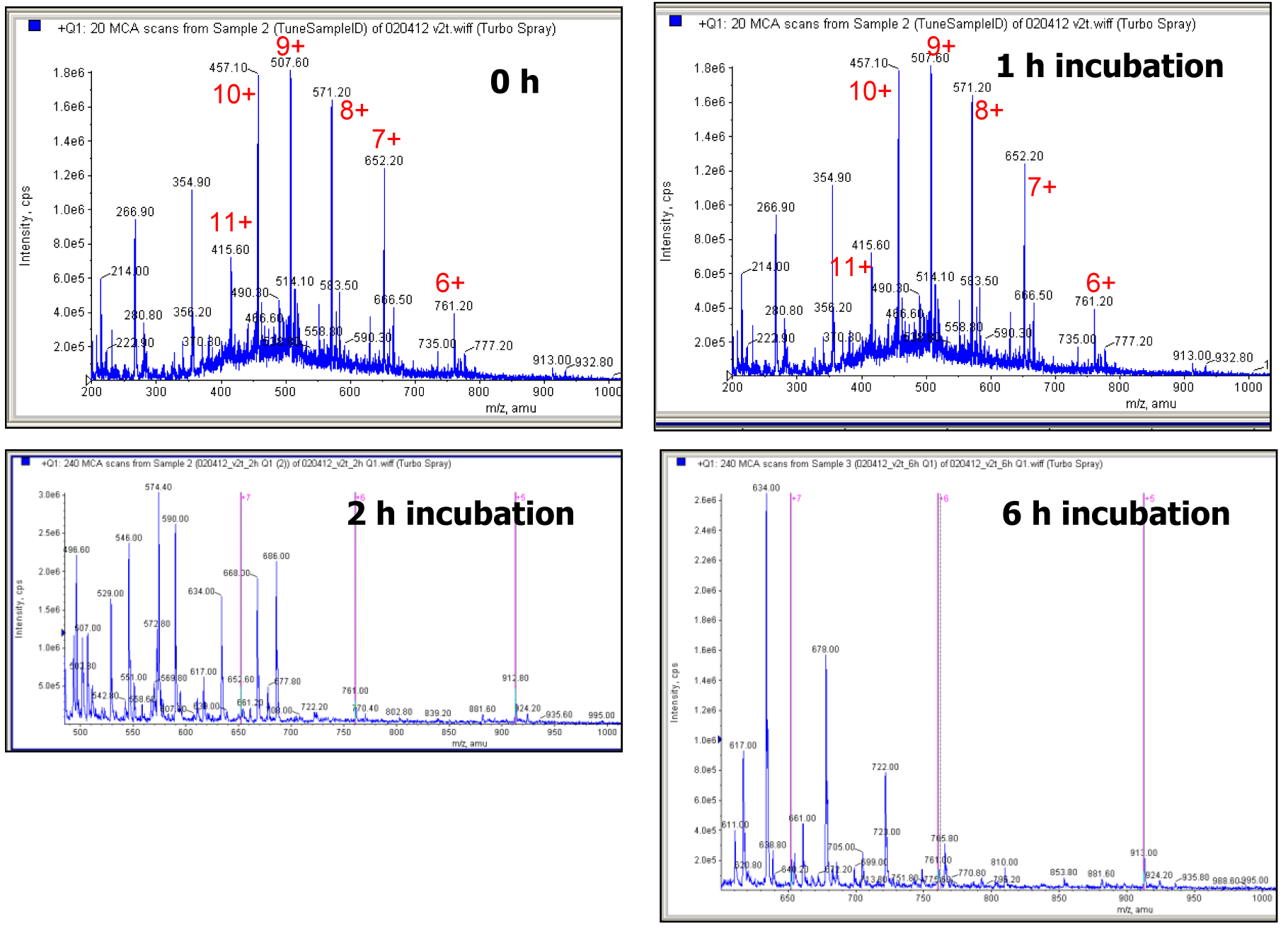

Supplement: Figure S2 — Positive charge electrospray ionization mass spectrometry of B4010 after incubation with trypsin (enzyme:B4010 = 1∶100). The multiple charged ions are shown in red color. The various multiple charged ions that correspond to B4010 are labeled in the figure. Note the progressive decrease in the B4010 ion peaks with incubation time. (TIF) [file pone.0087730.s002.tif]

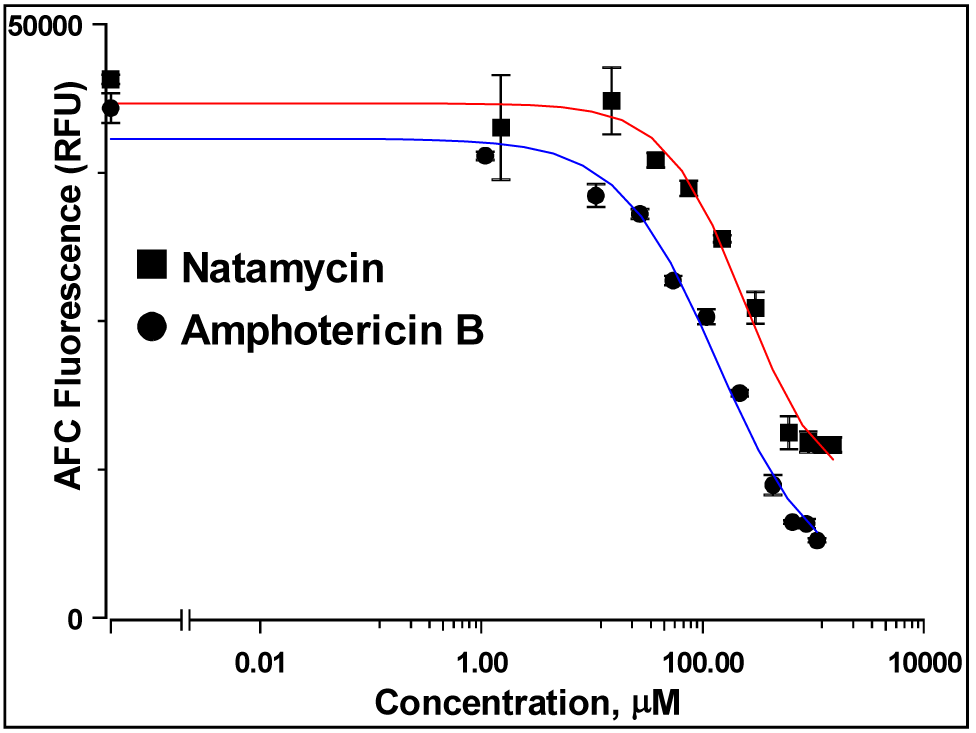

Supplement: Figure S3 — Cytotoxicity of polyene antifungals to HCE cells. The polyene antifungals were incubated with the cells for 24 h and the amount of intracellular ATP was quantified by AFC fluorescence. (TIF) [file pone.0087730.s003.tif]

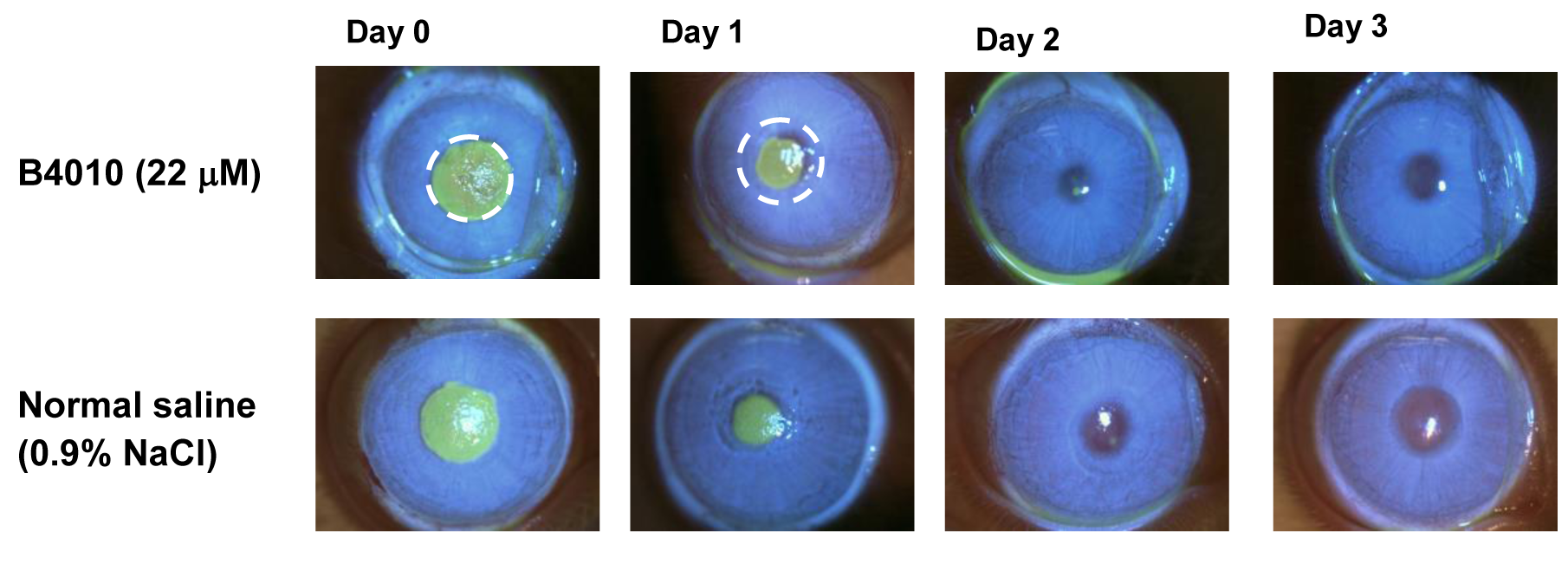

Supplement: Figure S4 — Fluorescein image of the representative cornea acquired at various time points after wound. The outer broken ring in the top panels represents original wound area. The re-epithelialization rate is identical for B4010 and saline, confirming the safety of B4010 in clinical settings. (TIF) [file pone.0087730.s004.tif]

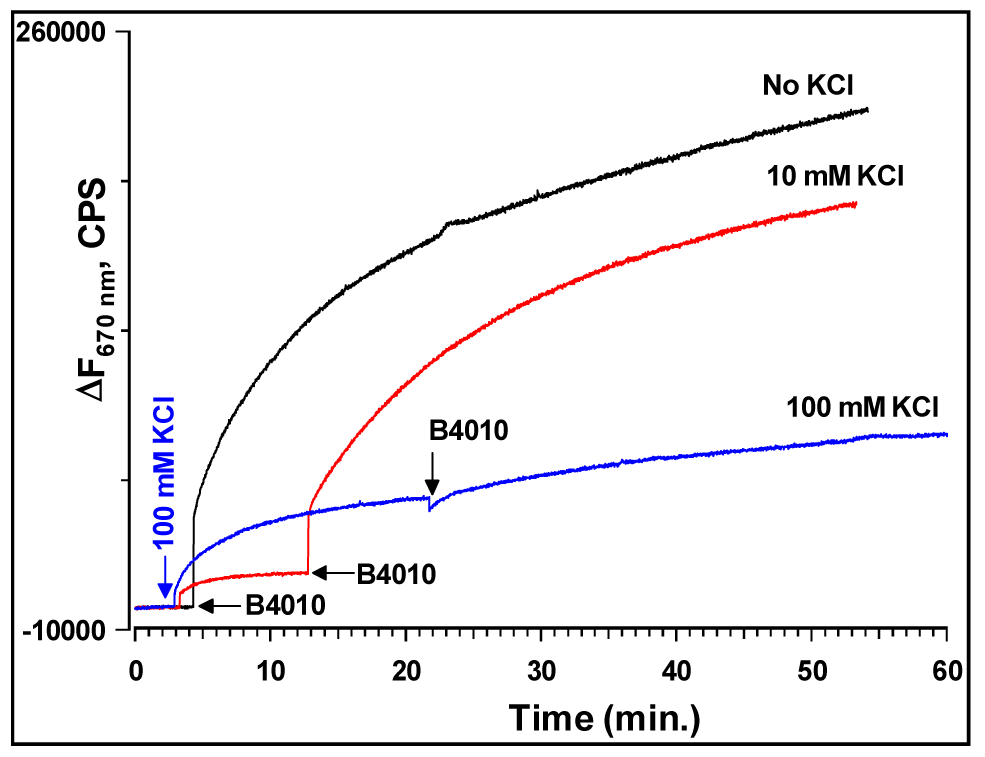

Supplement: Figure S5 — Effect of KCl on membrane potential of B4010 monitored by diSc3-5 fluorescence intensity at 670 nm. The blue arrow indicates time of addition of 10/100 mM KCl and the black arrows indicate time addition of B4010 (5.5 µM). Note that at higher concentration of KCl, the depolarization caused by B4010 became very weak. (TIF) [file pone.0087730.s005.tif]

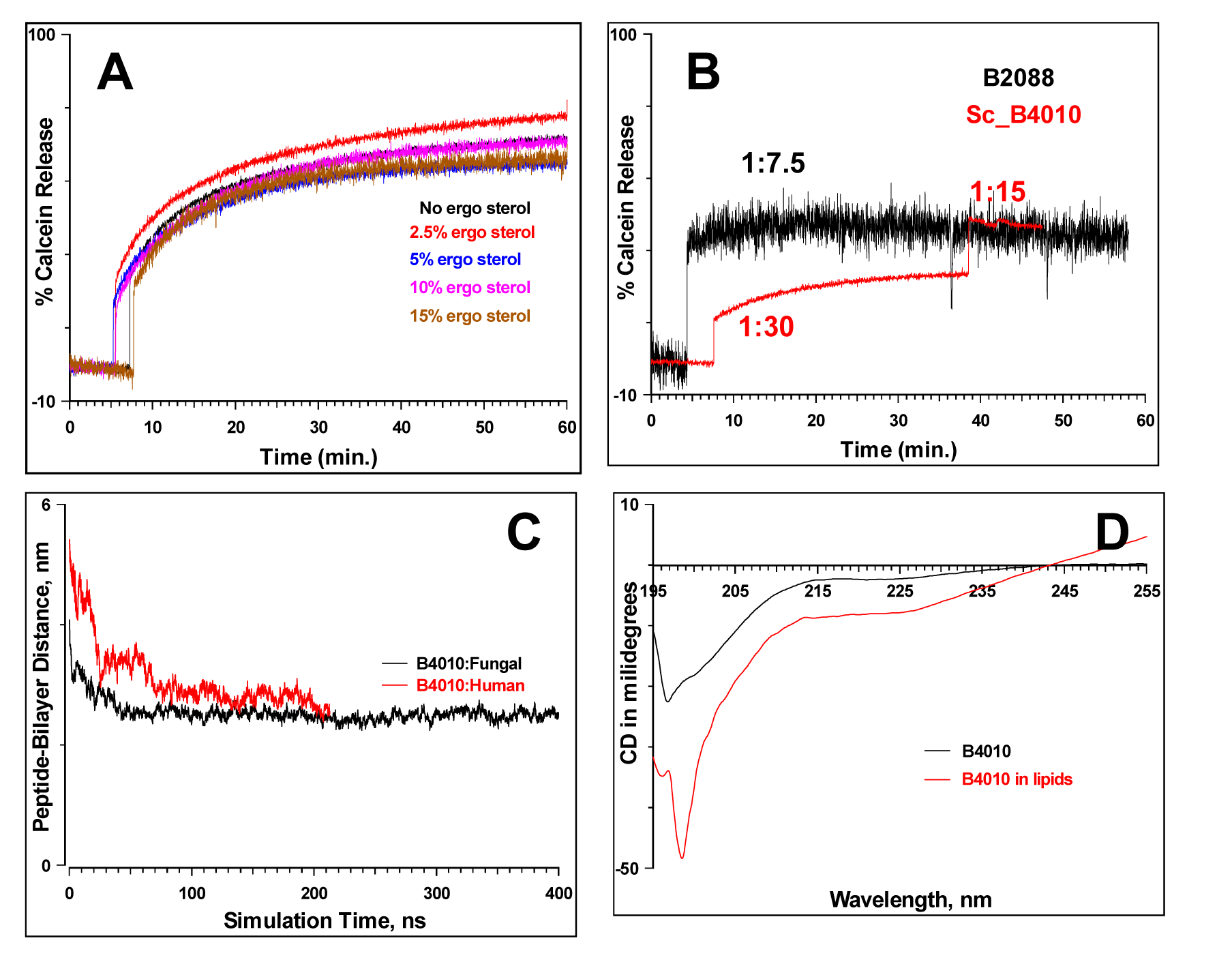

Supplement: Figure S6 — (A) Calcein leakage assay from SUVs containing various % of ergosterol. In all the cases the peptide:lipid molar ratio was 1∶15. (B) Calcein release assay from mixed liposome containing 15% ergosterol in the presence of B2088 and Sc_B4010. The concentration of B2088 was doubled to match the equivalence of tetrabranched peptides. Numbers indicate the peptide lipid ratio. (C) Time course of insertion of B4010 in fungal (black) and human (red) model membranes. The values of insertion are calculated as the difference in distance between centre of mass of B4010 and the bilayer center. (D) CD sepctra of B4010 in buffer and in model lipid containing 15% ergosterol. (TIF) [file pone.0087730.s006.tif]

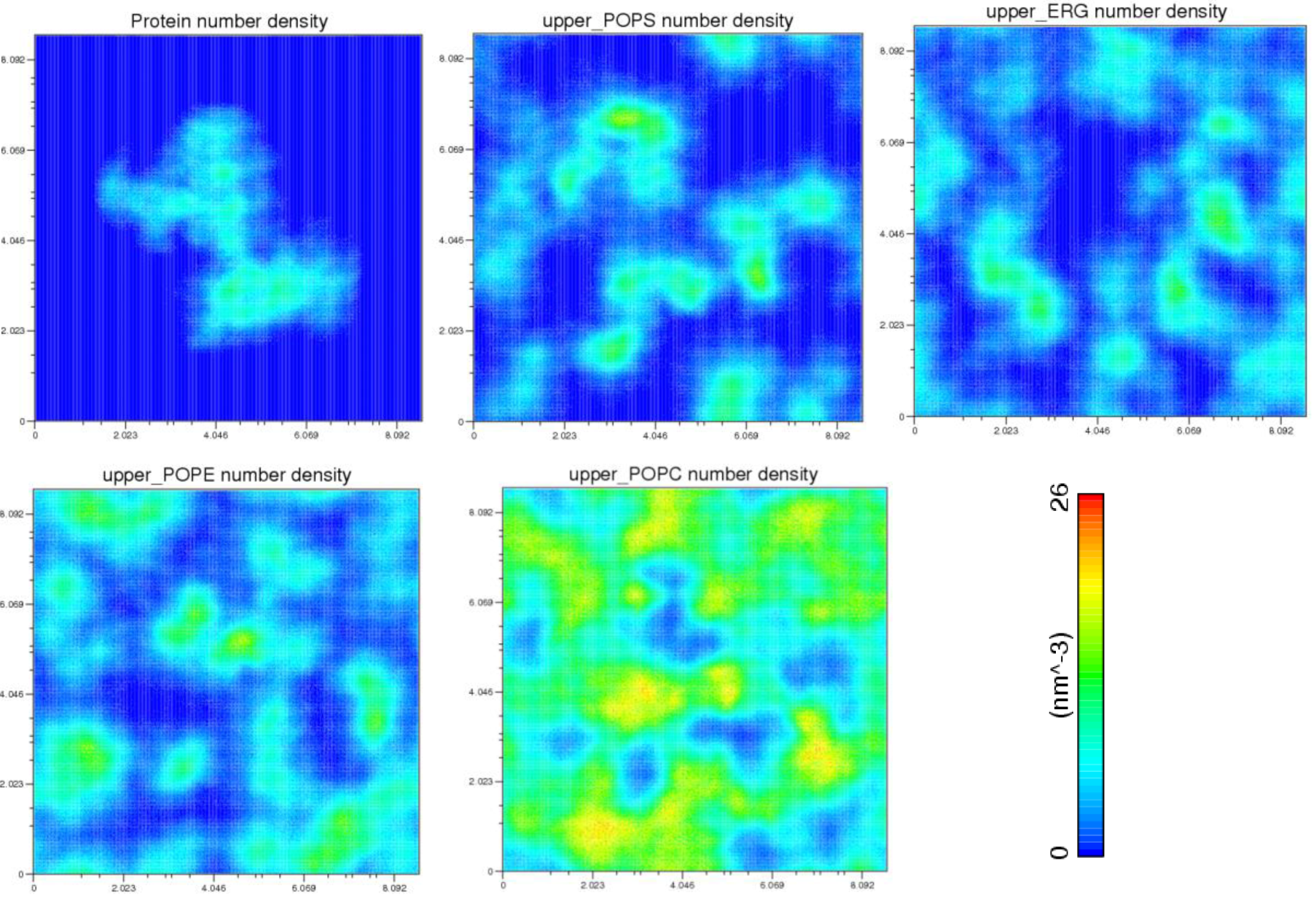

Supplement: Figure S7 — The 2-dimensional number density map of peptide and each type of lipids in the upper leaflet of the membrane based on the last 200 ns of a total 400 ns MD simulations. The simulations clearly show the preferential interaction between the POPS molecules and the preferential exclusion of ergosterol molecules with the peptide. (TIF) [file pone.0087730.s007.tif]

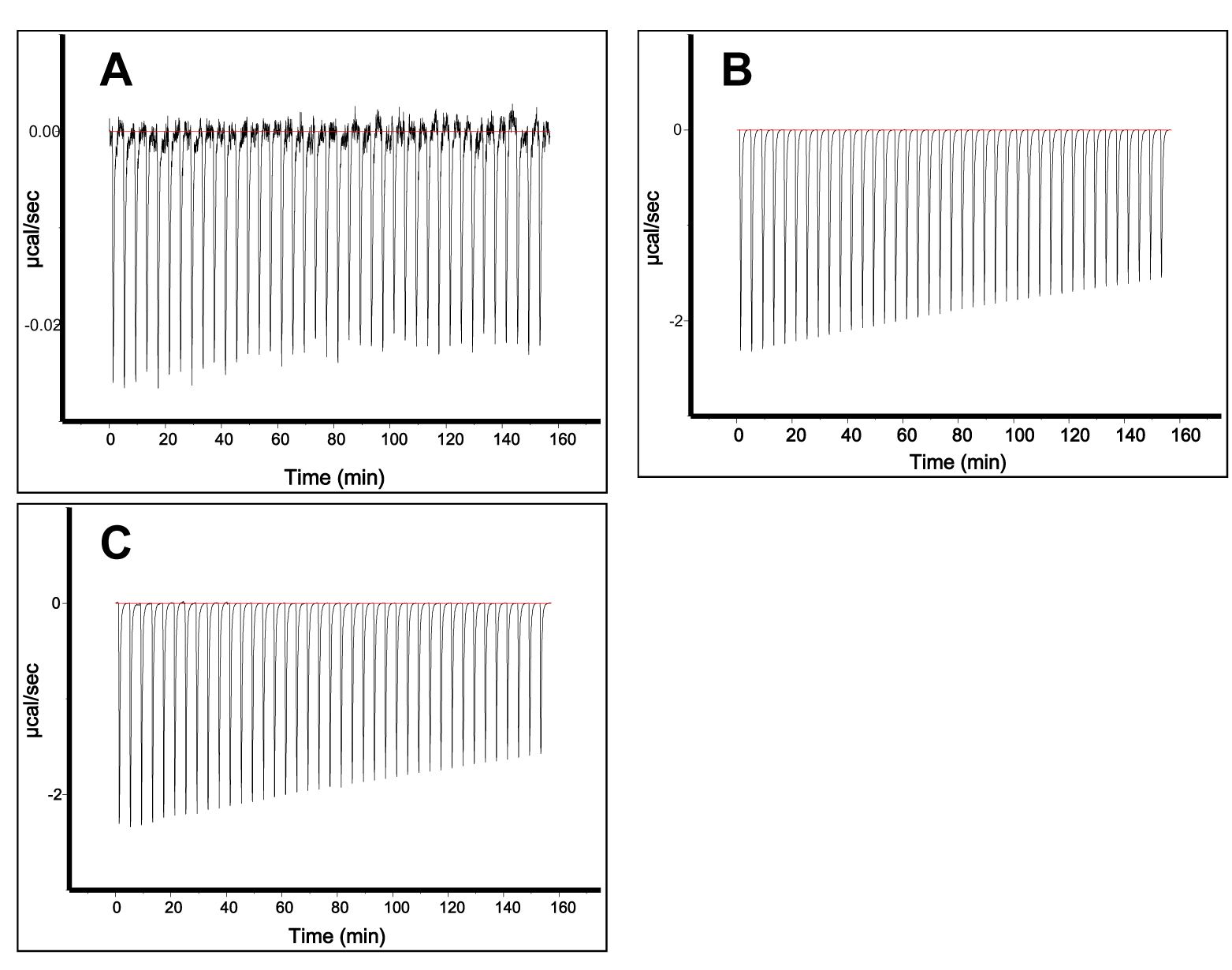

Supplement: Figure S8 — ITC heat flow traces (raw data) obtained by titrating PC SUVs containing 15% ergosterol to (A) B4010 (B) Amphotericin B and (C) Natamycin. Note that the peptide displayed very week interactions with the model lipid supporting the MD simulations results. However, the polyene antifungal amphotericin B showed pronounced heat changes upon interaction with ergosterol containing lipids. (TIF) [file pone.0087730.s008.tif]
